# Gender Equity Issues in Orthopaedics: A Scoping Review

**DOI:** 10.1007/s43465-025-01415-4

**Published:** 2025-05-26

**Authors:** Emily K. Schaeffer, Delaney R. Webber, Sharon Shrestha, Morgan G. Tidler, Tamseela Ahmed, Andrea M. Simmonds

**Affiliations:** 1 Department of Orthopaedic Surgery, BC Children’s Hospital, 4500 Oak St., Vancouver, BC V6H 3V4 Canada; 2 Department of Orthopaedics, The University of British Columbia, Vancouver, BC Canada

**Keywords:** Gender equity, Gender disparity, Orthopaedics, Representation, Discrimination, Gender bias, Women in orthopaedics, Scoping review

## Abstract

**Background:**

Patients have improved outcomes when their diversity is reflected in the healthcare team providing treatment. Despite recent increases in female representation, orthopaedics continues to lag behind other medical specialties. The purpose of this study was to identify major themes relating to gender equity in orthopaedics, examine trends in gender representation, and summarize the existing evidence within these themes.

**Methods:**

We conducted a scoping review in accordance with PRISMA guidelines using Medline, EMBASE and Global Index Medicus to identify original research articles on gender equity issues in orthopaedics. Data-driven studies examining issues across the orthopaedic training spectrum were included. A thematic analysis was conducted and descriptive statistics were used to describe trends within each theme.

**Results:**

In total, 179 studies were included representing seven themes: gender diversity and representation (*n* = 45), research and authorship (*n* = 41), leadership and mentorship (*n* = 30), microaggressions and lived experiences (*n* = 24), gender-based health impacts (*n* = 13), monetary aspects (*n* = 10), and geographic representation (*n* = 5), with 11 miscellaneous studies. Nearly 80% (*n* = 133) of studies originated from the USA, and 74.4% of studies were published from 2021 to 2025. Nearly all included studies reported that while there have been modest improvements in female representation, there remains significant gender disparity, and the field lags behind other specialties.

**Conclusion:**

This scoping review identified a compelling volume of literature demonstrating that women are underrepresented across all career stages in orthopaedics. Women surgeons disproportionately experience microaggressions, discrimination and health impacts. While representation is improving, concerted, collaborative efforts are needed to achieve gender parity in the field globally.

**Supplementary Information:**

The online version contains supplementary material available at 10.1007/s43465-025-01415-4.

## Introduction

The shifting global landscape makes diversity, equity and inclusion efforts more important than ever to ensure equitable access to optimal care for all patients, and workplace safety and well-being for all healthcare professionals worldwide. Despite long-time recognition of the lack of diversity in the field of orthopaedics, gender representation remains an issue that lags behind other medical specialties.^1^ Women now make up the majority of medical school graduates in North America (55.4% in the United States [USA]^2^ and 58% in Canada^3^), and the 2020–2021 All India Survey on Higher Education (AISHE) shows medical school enrolment reaching gender parity.^4^ However, this trend is not reflected in orthopaedic surgery worldwide, with only approximately 5–6% of orthopaedic surgeons being women.^5^ Consequently, there is still substantial work to be done to achieve gender parity in orthopaedics. This is an issue that permeates into both the well-being and career development of orthopaedic professionals, and the experience of patients receiving orthopaedic care. It is well-established that patients experience better outcomes in care when the treating healthcare teams reflect patient diversity. This can only be achieved through the promotion of inclusive training and work environments for individuals in occupations associated with patient care. The impact is particularly prevalent in orthopaedics as each year, in addition to elective orthopaedic surgeries, there are an estimated 234 million major orthopaedic trauma surgeries performed worldwide.^6^ Thus, inequalities in gender representation within orthopaedics is an issue that impacts an extensive proportion of the world’s population.

Orthopaedic surgery, both historically and currently, is the surgical speciality with the smallest proportion of women, even compared to other surgical specialties facing immense gender discrepancies such as neurosurgery and thoracic surgery. For example, in 2021, 16.7% of orthopaedic surgeons in the US were women, compared to 20.4% in neurosurgery and 30% in thoracic surgery.^7^ Furthermore, orthopaedic surgery has experienced a much slower increase in gender diversity as compared to other surgical specialties. Over a 4-year period between 2018 and 2021, orthopaedics experienced a minimal increase in female surgeons (+ 2%), especially compared to other specialties, such as general surgery, which experienced a more substantial increase in gender diversity (+ 6%).^7^

To increase the proportion of women entering the field of orthopaedic surgery, there must be an understanding as to what barriers exist that may dissuade them from making such a career choice. The potential influencing factors may range from structural inequalities in pay and research/authorship opportunities to lived experiences of discrimination and microaggressions, and even direct impacts on physical and mental health. Given the broad range of these impediments, a solution to this problem is guaranteed to be multifaceted. A scoping review allows for the analysis of a wide variety of elements contributing to challenges that women face when entering or working in the field of orthopaedic surgery. More specifically, the purpose of this scoping review is to identify the major themes relating to gender equity issues in orthopaedic surgery, determine trends in gender representation in the field, and summarize the existing findings within each of these emerging categories.

## Methods

The following scoping review complies with the PRISMA-ScR (Preferred Reporting Items for Systematic Reviews and Meta-Analyses extension for Scoping Reviews) guidelines.^8^ All peer-reviewed, original research articles were eligible for inclusion in this scoping review if they were a data-driven study (e.g. retrospective reviews of demographic/membership or surgical data, survey studies etc.), and if they centred around gender equity and diversity issues in orthopaedics. Systematic and scoping reviews were included, and no date restrictions were applied to the search. Articles of all languages were considered if an abstract was also available in English. Conference proceedings, abstracts, commentaries, editorials, and narrative reviews were excluded. Additionally, the articles were excluded if the main focus of the study was on medical school, or if the article focussed on multiple surgical fields.

We first conducted an informal literature search to guide the development of our search terms and search strategy. In consultation with a university librarian, we designed a search to capture all literature related to gender equity issues in orthopaedics. We used Embase, Medline and Global Index Medicus databases to conduct our search. Both MeSH terms and free-text keywords related to orthopaedics and gender diversity were used, as well as Boolean operators (AND, OR). The search strategy for Embase and Medline is shown in Table 1. The search strategy for Global Index Medicus is available in Supplemental Table 1.

**Table 1:** Search strategy for Medline and Embase

#	Term	Rationale
1	Orthopaedics/	MeSH terms are used to capture as many papers on orthopaedics as possible
2	(orthop?edic* or orthopod?).tw,kf	Other orthopaedic-related keywords are included to capture poorly indexed papers related to the topic
3	1 or 2	Step 3 is done to combine all orthopaedic related keywords and subjects
4	Women’s rights/ or gender equity/	MeSH terms are used to capture as many papers on gender equity as possible
5	((gender or wom#n or female or pregnancy) adj4 (bias or diversity or stigma or norms or leadership or autonomy or train? or training or competence or microaggression? or burnout or dropout? or equity or inequity or discrimination or equality or inequality or issue? or barrier? or inclusivity or inclusion or EDI or mentorship or representation or disparity or disparities or experience? or challenge? or compensation or authorship or work life balance or worklife balance or flexibility or skill? or pay or undervalue? or opportunit?)).tw,kf	Other gender equity-related keywords are included to capture poorly indexed papers related to the topic
6	4 or 5	Step 6 is done to combine all gender equity-related keywords and subjects
7	3 and 6	Step 7 looks for all orthopaedics-related articles, combined with the gender equity related keywords

After retrieving data from Embase, Medline and Global Index Medicus databases, articles were exported into Covidence for removal of duplicates, followed by title and abstract screening. Two independent reviewers assessed each title and abstract to determine if the articles met the inclusion criteria for full text review. In the case of disagreement, a third reviewer made the final decision on whether or not the abstract should be removed. The articles that passed initial title and abstract screening were then subject to full text review by two independent reviewers. Conflicts were resolved by a third reviewer.

Following full text review, included studies were exported onto a Google spreadsheet for data extraction and a thematic analysis was conducted, and those with a minimum of five representative studies were identified as themes. The data extraction sheet was developed and calibrated across reviewers, and data points included: article title, journal of publication, year of publication, gender of first author, gender of corresponding author, country article was published in, article theme, theme subcategory (if applicable), study type, sample size, primary outcome measure (if applicable), study purpose and main study findings. Data extraction was divided across reviewers according to theme. Author gender was inferred using a combination of the Genderize database^9^ and manual search for author information online.

There was marked heterogeneity across included studies relating to participant demographics, purpose and outcome measures. This heterogeneity, combined with the predominance of retrospective and survey study designs, prevented a formal meta-analysis and risk of bias assessment. We summarized our results using descriptive statistics. We used frequencies and proportions to present the study characteristics overall and by outcome in each theme.

## Results

### Study Selection

**Fig. 1:**
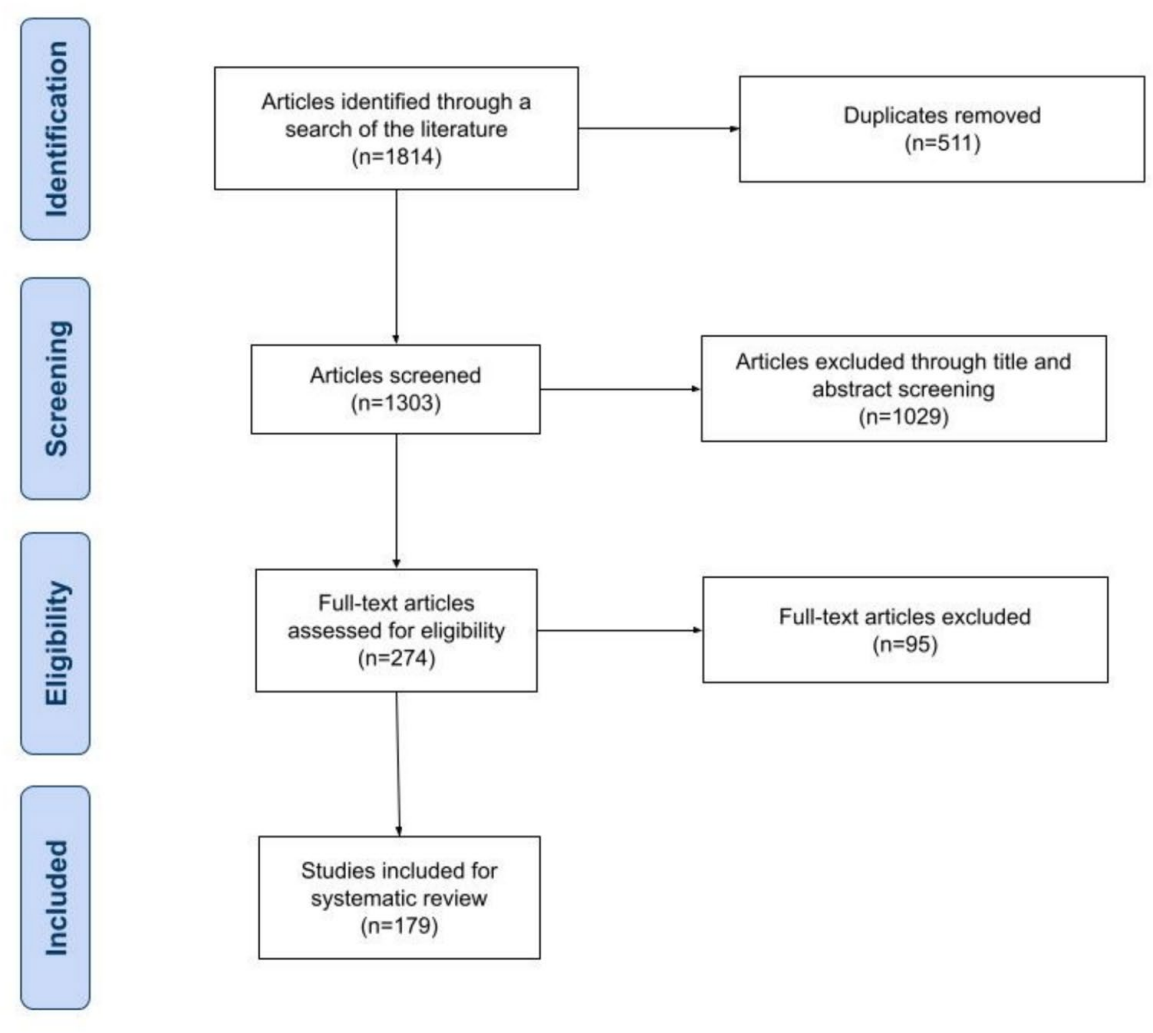
Preferred reporting items for systematic reviews and meta-analyses extension for scoping reviews (PRISMA-ScR) flow chart showing article selection for review

A total of 1814 articles were identified in the initial database searches. After removal of duplicates and title and abstract screening, 274 articles were selected for full text review, of which 179 met eligibility criteria (Fig. 1). We conducted a thematic analysis during full text review, identifying seven key themes: (1) gender diversity and representation (*n* = 45, 25.1%), (2) research and authorship (*n* = 41, 22.9%), (3) leadership and mentorship (*n* = 30, 16.8%), (4) microaggressions and lived experiences (*n* = 24, 13.4%), (5) gender-based health impacts (*n* = 13, 7.3%), (6) monetary aspects (*n* = 10, 5.6%), and (7) geographic representation (*n* = 5, 2.8%). Articles that did not fall under any of these themes were grouped into a Miscellaneous category (*n* = 11, 6.1%).

### Study Characteristics

**Fig. 2:**
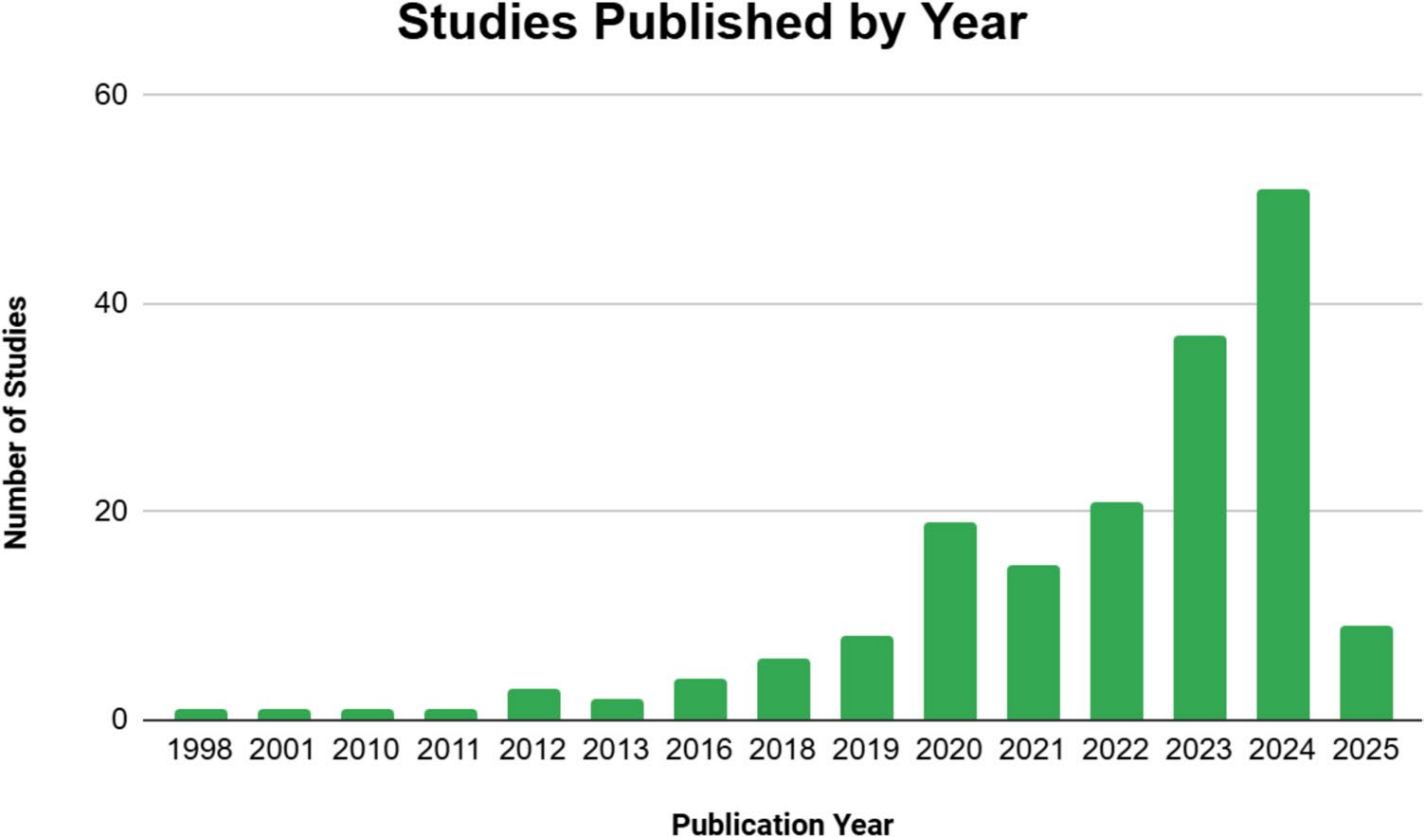
Distribution of included studies by publication year

**Fig. 3:**
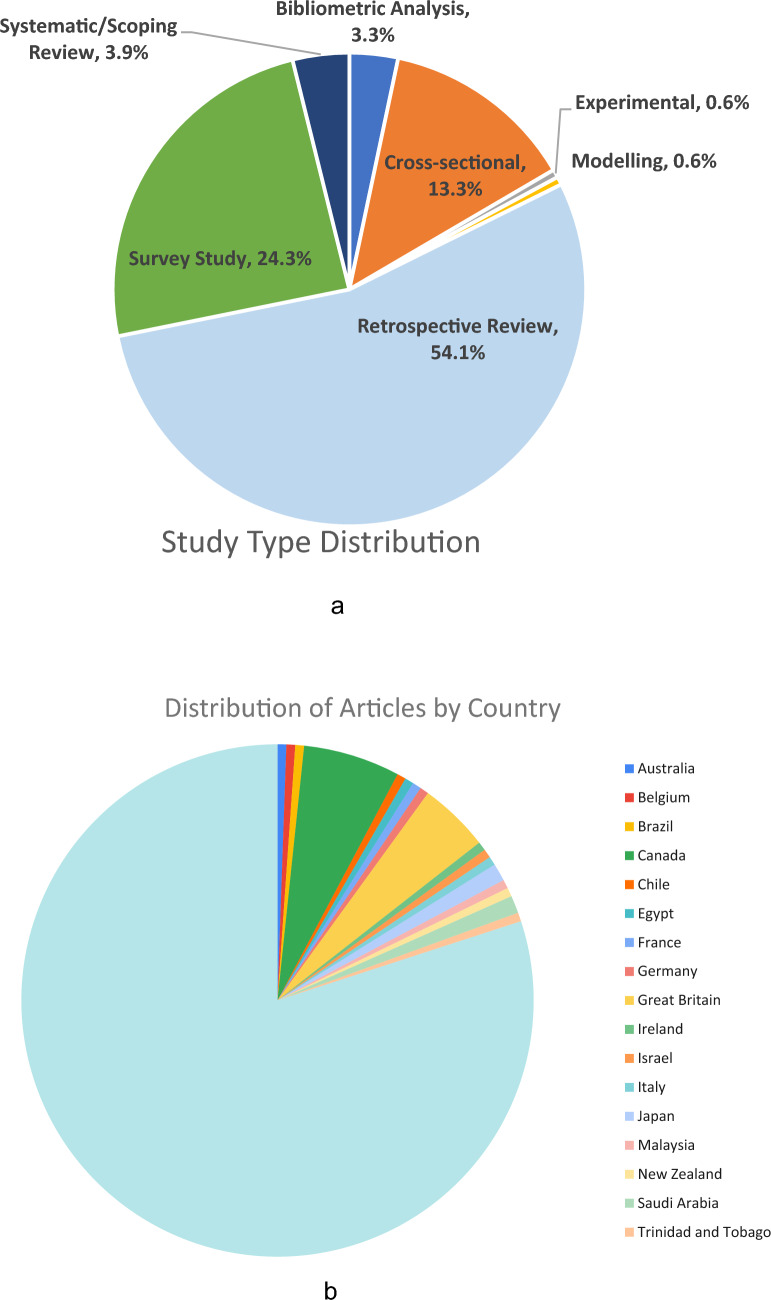
Distribution of included studies by **a** study design; and **b** country of origin

**Fig. 4:**
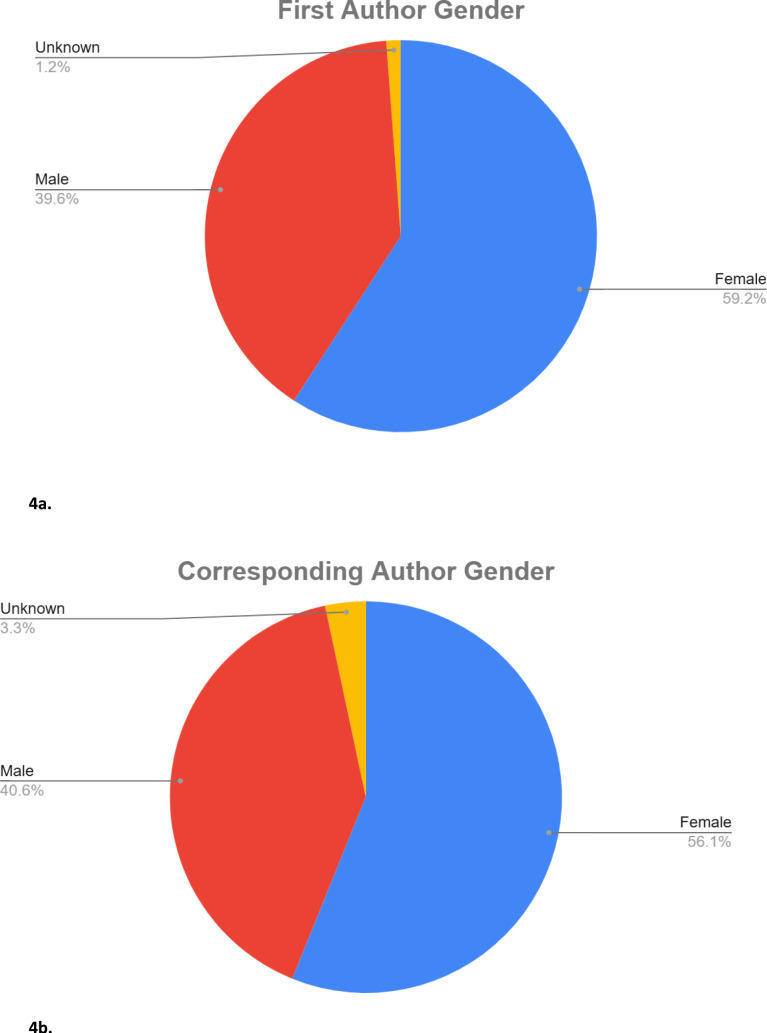
Percentage of female **a** first authors; and **b** corresponding authors

The 179 included studies were published between 1998 and 2025 (Fig. 2), with 74.3% (*n* = 133) published from 2021 to 2025. The majority of studies were retrospective reviews (*n* = 98, 54.1%), followed by survey studies (*n* = 44, 24.3%) and cross-sectional analyses (*n* = 24, 13.3%). The remaining studies comprised bibliometric analyses, systematic or scoping reviews, experimental and modelling studies (Fig. 3a). The majority of articles originated from the USA (*n* = 143, 79.9%), followed by Canada (*n* = 11, 6.1%) and Great Britain (*n* = 8, 4.5%). Fifteen other countries were represented with one or two studies each (Fig. 3b). Studies were published across 58 distinct journals, with Clinical Orthopaedics and Related Research and Journal of Bone and Joint Surgery being most prominent (*n* = 23 each, 12.8%) (Supplemental Table S2). Over half of both first authors and corresponding authors were female (Fig. 4a, b, respectively). Distribution of first and corresponding authors by theme is shown in Table 2.

**Table 2:** Female first and corresponding authors distribution by theme

Theme	Number of articles, *N* (%)	Female first authors, *N* (%)	Female corresponding authors, *N* (%)
Gender diversity and representation	45	*21	*21
Research productivity and authorship	41	25	**18
Leadership and Mentorship	30	18	18
Microaggressions and lived experiences	24	19	17
Monetary aspects	10	5	6
Gender-based health impacts	13	*11	*11
Geographic representation	5	1	2
Miscellaneous	11	10	7
TOTAL	**179**	**110**	**100**

Bold value indicates the overall total across all themes

*Gender of 1 author was unknown

**Gender of 4 authors were unknown

### Gender Diversity and Representation

Studies broadly examining gender diversity and representation in orthopaedic surgery were most prevalent (*n* = 45). The characteristics of the studies included in this theme are detailed in Supplemental Table S3. Most (*n* = 41, 91.1%) originated in the USA, and 71.7% (*n* = 32) were retrospective reviews. In total, 12 studies examined aspects of gender diversity and female representation in residency programmes in the USA (*n* = 10) and Canada (*n* = 2).^S1−S12^ Five of these studies, published from 1998 to 2025, all noted that while representation of women in residency is increasing, orthopaedics still substantially lags behind other surgical and non-surgical specialties,^S1−S5^ and during residency, women trainees have higher attrition rates than men.^S6^ Specifically, Biermann et al. (1998) reported the proportion of female orthopaedic residents increased from 1.2% in 1977 to 7.1% in 1996, while the percentage of all women residents in that time period increased from 13.5% to 34%.^S1^ Likewise, Wang et al. (2023) reported a 9.2% increase in female orthopaedic residents from 2001 to 2020; however, that time period saw an aggregate increase of 16.3% female representation across all surgical specialties.^S4^ Two studies noted that trainee gender diversity was a reflection of faculty gender diversity, and correlated with programme ranking.^S7,S8^

Similar trends were seen across 15 studies examining orthopaedic fellowship programmes (14/15 from the USA): three generally,^S13−S15^ four in Trauma,^S16−S19^ three in Arthroplasty,^S20−S22^ two in Foot and Ankle,^S23,S24^ two in Sports Medicine,^S25,S26^ and one in Spine.^S27^ The lack of women representation also persisted at the Faculty level generally, despite modest increases over time.^S28−S34^ For example, Shah et al. (2020) found that while women orthopaedic faculty increased by 8.8% from 1997 to 2016, other specialties saw growth of 13.9%. Additionally, the proportion of women in senior faculty positions grew even more slowly (7.3% vs. 14.7%). However, one study did find that women had higher than expected representation at each academic rank in comparison to their relative membership representation in the American Orthopaedic Association (AOA).^S34^ Specifically, participation-to-prevalence ratios (PPR) of women in assistant, associate, and full professor positions were 1.45, 1.56 and 1.27, respectively.^S34^ Underrepresentation of women in faculty or clinical practice was also apparent in studies focussing on Arthroplasty,^S35−S38^ Sports Medicine,^S39,S40^ Spine,^S41^ and Foot and Ankle^S42^ specialties. Overall, several studies found decreasing representation of women at each stage of the training pipeline.^S43−S45^ One modelling study predicted if current diversity efforts and programme remain in current state, a steady state of 25% women representation in orthopaedic trainees is expected to be reached by 2055.^S12^

### Research and Authorship

Research and Authorship was the next most prevalent theme (*n* = 41), and characteristics of the included studies are detailed in Supplemental Table S4. Again, the USA was the dominant country of origin, representing 80.4% of studies (41), and 63.4% (*n* = 26) were retrospective reviews. In total, 12 studies reported on women representation as speakers, moderators or panellists at conferences (11),^S46−S56^ or Grand Rounds (1).^S57^ Most of these studies noted that while representation of women as speakers/moderators/panellists had increased over time,^S46−S48^ they were still markedly outnumbered by men.^S49−S53^ For example, Mencia et al. reported a steady increase in the number of female presenters at the annual meetings of the Caribbean Association of Orthopaedic Surgeons over a 5-year period (14.3%–24.4%); however, men still made up the majority (80.5%) of presenters.^S48^ While one study reported lower proportion of women as speakers/moderators/panellists in comparison to proportion of society membership,^S54^ two other studies noted the opposite trend.^S55,S56^

Twenty-six studies examined authorship of orthopaedic publications. The majority (*n* = 24) examined gender trends in first and last authorship: 13 across orthopaedics in general,^S58−S70^ three in paediatrics,^S71−S73^ two in arthroplasty,^S74,S75^ two in Sports medicine,^S76,S77^ one in Trauma,^S78^ one in Foot and Ankle,^S79^ one in Oncology,^S80^ and one in Shoulder and Elbow.^S81^ All studies reported continued significant underrepresentation of women as both first and senior authors, despite modest to substantial increases over time. This held true across journals and specialties. Specifically, the reported proportion of female first authors ranged from 0 to 19.9% and the proportion of female senior authors ranged from 0 to 17.8% across studies.^S58−S81^ The remaining two studies on authorship examined gender differences in publication volume of orthopaedic residents, with both noting men having substantially more publications than women.^S82,S83^ Specifically, Cho et al. examined 178 residency programmes and found that females were first author on 22% of publications, despite representing 31.8% of the residency programmes.^S83^ The final three studies reported that women were substantially underrepresented as principal investigators (PIs) on clinical trials,^S84,S85^ and members of research consortiums.^S86^ For example, Silvestre et al. examined female representation in a sample of 157 clinical trials in arthroplasty, and found that of 192 involved PIs, only two PIs were female (1%).^S84^

### Leadership and Mentorship

Characteristics of the 30 included studies in the Leadership and Mentorship theme are detailed in Supplemental Table S5. Twenty-five studies (83.3%) originated in the USA and 60% (*n* = 18) were retrospective reviews. Overall, eight studies pertained to the influence of gender representative mentorship or role models in residency and fellowship choices. Most reported a positive impact of having a female mentor during training at an individual or programme level.^S87−S92^ For example, Ranson et al., found that there was a positive correlation (r = 0.577, *α* < 0.001) between the female residents and the women faculty at 208 accredited orthopaedic surgery residency programmes across the US.^S87^ However, two studies refuted that view.^S93,S94^ Specifically, Hill et al. administered a survey to 244 female academic orthopaedic surgeons, less than 23% of whom mentioned a female mentor positively influenced them at various levels of training.^S93^ Underrepresentation of women in faculty leadership positions such as Chairs, Program Directors or Fellowship Director was apparent across all 11 studies reporting on this issue.^S94−S104^ Particularly, Proal et al., reported a total of only four female (8.2%) Division Chiefs out of the 49 US shoulder and elbow Division Chiefs.^S103^ Similarly, 10 studies focussed on gender representation in Orthopaedic Society Leadership found persistent underrepresentation in comparison to society membership.^S105−S114^ For example, Poon et al. examined the membership and leadership positions of POSNA members from 2014 to 2018, discovering that while women membership steadily increased from 18 to 22%, the percentage of Council Chair Positions held by women did not (7%–12%).^S105^ Of the two remaining studies, one found gender representation on journal editorial boards was commensurate to representation across academic orthopaedics.^S115^ The other study found marked underrepresentation of women surgeons in professional sports (1.6%).^S116^

### Microaggressions and Lived Experiences

Characteristics of the 24 included studies in the Microaggressions and Lived Experiences theme are detailed in Supplemental Table S6. While 66.7% (*n* = 16) of studies were from the USA, six other countries were represented in this theme (Australia, Brazil, Canada, Great Britain, Malaysia, and Saudi Arabia). The majority (*n* = 20, 83.3%) were survey studies examining experiences and perceptions, and 23/24 studies were published since 2020. Sexual harassment and discrimination were highlighted as disproportionately impacting women orthopaedic surgeons and trainees in 9/13 studies that focussed on gender bias or microaggressions.^S117−S125^ Specifically, Giglio et al. and Halim et al. reported 97.5% and 81% of their survey respondents, respectively, had experienced microaggressions or gender-based harassment.^S117, S122^ Of the remaining four studies, one found no gender bias in evaluator ratings of trainee arthroscopy skills,^S126^ but another did find women trainees were at risk of a competency bias, rating themselves lower than their evaluators and male counterparts.^S127^ Women surgeons were also subject to gender bias from patients, as women who wore feminine business attire instead of scrubs were perceived as less capable.^S128^ Finally, 60% of orthopaedic department websites use gendered language to describe leadership positions.^S129^ Barriers and facilitators for women in orthopaedics were examined in one scoping review ^S130^ and six survey studies.^S131−136^ Negative stereotypes, male-dominated culture, gender bias, discrimination and harassment were identified as key barriers women routinely face in orthopaedic training and careers. Liew et al. found that the top barriers for women going into the orthopaedics specialty were due to physical strength (56%), discrimination from the public (52%), and gender discrimination in terms of career opportunities (28%).^S133^ Three studies explored general lived experiences for women surgeons, identifying disproportionate workplace conflict, mistreatment and inequality as adverse factors.^S137−S139^ For example, Gerull et al. noted that 84% of female residents were subject to various types of mistreatment such as physical, verbal, or sexual abuse, which was significantly higher than the number of men (43%) who reported mistreatment in similar areas (*p* < 0.001).^S137^ Finally, one study highlighted the increased experience of workplace violence for women from patients/families and coworkers.^S140^

### Gender-Based Health Impacts

The characteristics of the 13 included studies in the Gender-based Health Impacts theme are detailed in Supplemental Table S7. USA (*n* = 8), Canada (*n* = 2), Great Britain (*n* = 2) and Israel (*n* = 1) were represented, and 9/13 studies were surveys. Nine studies focussed on pregnancy and fertility,^S141−S149^ highlighting the increased rate of difficulties women surgeons experience, and the necessity to delay starting a family due to work demands. Across four studies, an average of 58% of female orthopaedic trainees and surgeons reported deferring childbirth.^S142,S143,S145,S146^ This rate is consistent with two systematic reviews that reported intentional deferral of childbearing by up to 67%^S148^ and up to 67.3%^S141^ of female orthopaedic surgeons during residency. Three studies identified the prevalence of burnout amongst women orthopaedic surgeons.^S150−S152^ Hiemstra et al. found that 50.5% of female orthopaedic surgeons experienced burnout, and Carter et al. found women were more likely to experience both personal (57%, *p* < 0.001) and team burnouts (57%, *p* < 0.001) than men (30%, 43.2%).^S150,S151^ Finally, one study reported access to and use of female-specific lead in the operating room is insufficient.^S153^ This survey study, conducted by Mengers et al., found the majority of respondents to report female- specific lead was either unavailable (45%) or available in insufficient quantities (5.5%) at their institution.^S153^

### Monetary Aspects

The characteristics of the 10 included studies in the Monetary Aspects theme are detailed in Supplemental Table S8. Studies predominantly originated from the USA (*n* = 9, 90%) and 90% were retrospective reviews. Of these, five studies highlighted issues of compensation, reporting the substantial wage or billing gaps seen between women and men surgeons.^S154−158^ Robin et al. highlighted that among the 347 highest-compensated orthopaedic surgeons, only one of them was a woman. ^S154^ A retrospective review conducted by Beebe et al. highlighted that male surgeons had higher incomes than female surgeons despite working equivalent hours ($802,474 vs. $560,618, *p* < 0.001).^S156^ Additionally, three studies found this gender gap exists in industry partnership and payment opportunities.^S159−S161^ Forrester et al. found the median payment for men surgeons was greater than for women surgeons (USD 1027 vs. USD177, *p* < 0.001).^S159^ Ray et al. found the 99.6% of royalties and consulting payments were made to men, and male gender to be a predictor of the total number of payments (*β* = 5.12, *p* < 0.001).^S160^ Finally, two studies examined research grant funding; one found the majority of grant funds received were for studies with male Principal Investigators.^S162^ In contrast, the second found that Orthopaedic Research Excellence Fund resident grant recipients had greater representation of women compared to the national cohort of residents.^S163^

### Geographic Representation

The characteristics of the five included studies in the Geographic Representation theme are detailed in Supplemental Table S9. All studies were from the USA and 4/5 were retrospective reviews. One study found an association between hometown and undergraduate training locations to residency programme location match.^S164^ Cox et al. found 41% (308/744) of residents to match in the same division as their hometown and 40% (536/1329) to match to the same division as their undergraduate institution.^S164^ The remaining four studies found discrepant geographic representation of women in residency programmes across the USA.^S165−S168^

### Miscellaneous

The remaining 11 studies are described in Supplemental Table S10. Gender differences in competency were described in three studies,^S169−S171^ with one finding female trainees had a 26% increased risk of a non-standard competency review.^S169^ The other two studies found lack of women trainees was not due to academic merit differences,^S170^ or gender bias in application review.^S171^ Male trainees were found to have increased operative autonomy compared to female trainees in three studies from New Zealand, Great Britain, and Ireland.^S172−S174^ McColgan et al. showed that men had 145% increased odds of performing an operation with autonomy compared to women. ^S174^ Three survey studies probed career plan differences between women and men trainees, to inform workforce planning and better understand gender-specific motivations and influences.^S175−S177^ For example, Hariri et al. (2011) found that significantly more women than men (64% vs. 32%) planned a subspecialty-only practice, as well as to reduce work hours (65% vs. 47%) at some point in their career.^S175^ One study reported that women trainees had higher vulnerability to stereotype threat, impacting career progression and well-being.^S178^ Finally, one study reported that while women received awards from orthopaedic societies comparable to their membership representation, they were more likely to receive awards in diversity or education than in leadership.^S179^

## Discussion

Gender disparity in orthopaedics is a long-standing problem that has been recognized and acknowledged by orthopaedic societies globally. Despite more recent efforts to mitigate negative consequences and promote diversity, substantial work remains to be done. This scoping review highlights the wide-ranging impacts of gender equity issues across the orthopaedic landscape, identifying seven major themes that span from underrepresentation of women at all training levels and geographic regions, authorship and research involvement, leadership, mentorship, and compensation, to experiences of microaggressions, gender bias, workplace conflict, and gender-based health impacts such as pregnancy and burnout. The consequences of these issues are pervasive, impacting women throughout their careers from residency applications through senior faculty and society leadership roles.

The most prevalent finding across all the themes was clear: while efforts to increase gender diversity in orthopaedics have resulted in increased representation of women across many levels, there remains a substantial gap between orthopaedics and other medical specialties. This was evidenced by modest increases in women trainees in orthopaedic residency programmes,^S1−S5^ fellowships^S13−S27^ and faculty positions,^S28−S34^ but still falling far short of gender parity. Modelling studies indicate that with current engagement and diversity efforts, the USA would reach a steady-state of 25% women representation among orthopaedic trainees by 2055,^S12^ while Canada would achieve gender parity by 2060.^S5^ These estimates are relatively promising, but rely heavily on the sustainability of diversity efforts. Additionally, they are specific to a North American setting, and not representative of the global orthopaedic community.

Discrepant representation of women between orthopaedic subspecialties provides areas for shared learning and improvement. Women in paediatric orthopaedics were noted to have strong fellowship match rates,^S13^ first authorship rates,^S71^ fellowship director representation,^S98^ and panel representation at society conferences.^S51^ Additionally, women trainees expressed a preference for the paediatric subspecialty when making career choices.^S176,S177^ In contrast, spine surgery notably lacks women representation in fellowship,^S13,S27^ faculty,^S41^ and conference panels.^S51^ Understanding different approaches to recruitment and retention initiatives across different subspecialties can help to improve practices, sustain momentum and unify efforts globally.

Consistent trends were observed that female representation decreased at each stage of the orthopaedic training pipeline from medical school to residency to fellowship to faculty. Thus, there is a distinct lack of women in senior faculty positions. This has multifaceted consequences at both an individual and institutional level. Individually, lower representation of women in senior faculty may correlate with disproportionately low rates of senior authorship in the orthopaedic literature,^S58,S64,S67,S68^ in addition to creating barriers to women attaining society leadership positions^S105,S108,S109^ and grant funding.^S84,S85^ Institutionally, several studies noted that programmes with more women faculty attracted more resident applications from women. Consequently, a lack of women in senior faculty positions may hinder ongoing and future efforts to improve gender diversity in orthopaedic training. Female trainees in programmes with gender diverse faculty have more ready access to female mentors and role models, noted to be important in several studies.^S7,S8,S87,S88^

Despite distinct underrepresentation of women in orthopaedic training, programme and society leadership, and authorship, women orthopaedic surgeons are strikingly overrepresented in experiences of microaggressions, gender bias, discrimination and workplace conflict or violence.^S117−S140^ Most studies examining these issues were survey studies and are therefore subject to potential responder bias. However, every study found high prevalence of gender-based discrimination and harassment, with women overwhelmingly being the victims at the hands of both patients and families, and male colleagues. Additionally, both data-driven and experiential studies identify high rates of pregnancy and fertility complications for women in orthopaedics,^S141−S149^ causing many to delay or abstain from starting a family. Combined, consistent exposure to all of these negative experiences and barriers can lead to burnout, anxiety and depression affecting women at a higher rate than men.^S150−S152^ This can impact attrition rates across career stages, and further exacerbate the gender gap in representation, compensation and leadership.

The high volume of studies included in this scoping review emphasizes the importance of gender equity and diversity issues in the field of orthopaedics. Recognition of this has gained particular prominence in recent years, evidenced by nearly three quarters of all included studies being published since 2021. However, the earliest study identified was published in 1998,^S1^ demonstrating awareness of its significance for at least the last 27 years. Despite this, numerous included studies noted similar results year after year, with only modest gains seen in representation of women in orthopaedics. Several comprehensive studies examined and identified barriers and facilitators that women face in orthopaedic careers,^S130−S136^ but most facilitators have yet to be widely implemented in a systematic way at a larger than institutional level. Additionally, evaluation of the implementation of diversity programmes is lagging.

Programs like the Perry Initiative have been designed specifically to provide early opportunity and access to orthopaedics for female medical students in the USA.^10^ The initiative is seeing success in their approach, with participants almost uniformly reporting positive impacts on their perceptions of orthopaedics and their intentions to pursue an orthopaedic residency. More recently, Women in ORTHopaedics (WORTH), inspired by the Perry Initiative, was started in Canada, and has seen early positive impacts on female and gender diverse high school students.^11^ The approach of these programmes to provide early exposure and access to mentorship presents a potentially sustainable and broadly applicable approach to promoting and improving diversity in orthopaedics.

It should be noted, however, that the vast majority of literature in this area originates in the USA, comprising nearly 80% of all included studies. Most of the rest of the represented countries were also in the Global North. Women are typically even more underrepresented in various regions of the Global South, and therefore, initiatives to improve gender diversity must have global relevance and be able to adapt to context-specific barriers. For example, despite India achieving gender parity in medical school enrolment since 2020,^4^ women only represent an estimated 1% of orthopaedic surgeons in the country.^12^ Recently, efforts have been made to understand what limits female medical students in India from pursuing orthopaedics^13^; this is a first step to progress. It is, however, also important to provide opportunity for leadership, access to role models, and visibility for women pioneers already in orthopaedics in these underrepresented regions. Initiatives like WOICE (Women Orthopaedic surgeons in India Collective Empowerment)^13,14^ and WAVES APOA (Women’s Advocacy, Education and Support; Asia Pacific Orthopaedic Association)^15^ are gaining momentum and providing impetus to further diversity efforts.

Findings from this review provide multiple avenues for future research. First, surveying programmes that have been successful at increasing representation of women will help to identify effective approaches that can be implemented more broadly. Second, we must think beyond proportional representation as the primary measure of success. Future research should evaluate whether interventions and diversity programmes help to address systemic issues, and decrease experiences of discrimination, microaggressions, gender bias, and gender-based health impacts. Effective evaluation will allow for optimized strategies and continuous evolution with generational workforce changes.

There are several limitations to this scoping review. First, not all relevant articles may have been identified by the search strategy. However, given the substantial number of studies included, additional studies would be unlikely to alter the conclusions. Second, included studies were predominantly from the USA and other Global North countries, limiting our understanding of the applicability of the identified themes to regions in the Global South. The comprehensive search strategy utilized suggests this is likely due to a lack of literature available on this topic from these regions and points to an area of future study. Third, the evidence level was limited, with most included studies being of retrospective or survey design. Fourth, we focussed our scoping review on primary research studies to gain a comprehensive, quantitative view of themes surrounding gender equity in orthopaedics. We recognize other sources, such as commentaries, editorials, news items and conference symposia may provide other valuable avenues for insight on these topics.

In conclusion, this scoping review identified seven major themes pertaining to gender equity in orthopaedics: gender representation in orthopaedic programmes, research and authorship, leadership and mentorship, microaggressions and lived experiences, gender-based health impacts, monetary aspects, geographic representation. Women are consistently underrepresented across all career stages and areas, but disproportionately suffer the consequences of microaggressions, discrimination and health impacts.

Based upon these findings, we recommend continuing to promote and expand upon programmes such as the Perry Initiative, WORTH, WOICE, and WAVES APOA as an important step to increase female representation and empowerment in Orthopaedics. Additionally, supporting women to reach senior faculty positions, programme and society leadership roles, and lead authorship will help strengthen visibility of women in Orthopaedics, and promote opportunities for mentorship and sponsorship in the younger generations. The programmes providing early exposure for students, mentorship matching programmes for residents, fellows and junior faculty may be effective approaches to promoting and sustaining these improvements. Additionally, fostering male colleague allyship through awareness and education is critical. Overall, representation of women in orthopaedics is improving with modest increases over the last two decades, but sustained, systemic efforts will be required to continue moving toward gender parity globally.

## Supplementary Information

Below is the link to the electronic supplementary material.

Supplementary file1 (DOCX 15 KB)

Supplementary file2 (DOCX 24 KB)

Supplementary file3 (DOCX 86 KB)

Supplementary file4 (DOCX 40 KB)

## References

[1] Van Heest, A. (2020). Gender diversity in orthopedic surgery: We all know it’s lacking, but why? *Iowa Orthopaedic Journal,**40*, 1–4. 32742201 PMC7368510

[2] Clark, M., Kerslake, S., Bøe, B., & Hiemstra, L. A. (2024). Being a woman and an orthopaedic surgeon—a primer on the challenges we face. *Journal of ISAKO,**9*, 449. 10.1016/j.jisako.2024.05.008 38777119

[3] Burton, K. R., & Wong, I. K. (2004). A force to contend with: The gender gap closes in Canadian medical schools. *CMAJ,**170*, 1385–1386. 15111465 10.1503/cmaj.1040354 PMC395806

[4] Government of India Ministry of Education. All India Survey on Higher Education. New Dehli: Department of Higher Education; 2021–2022.

[5] Green, J. A., Chye, V. P. C., Hiemstra, L. A., Felländer-Tsai, L., Incoll, I., Weber, K., Pohl, M., Kollias, C., Maasalu, M., Iñiguez, M., Bytyqui, D., Fok, M., Liverneaux, P., Hamdan, E., Lupondo, V., & Hing, C. B. (2020). Diversity: Women in orthopaedic surgery—a perspective from the International Orthopaedic Diversity Alliance. *JTO.,**08*(1), 44–51.

[6] Dobson, G. P. (2015). Addressing the global burden of trauma in major surgery. *Frontiers in Surgery [Internet].,**2*, 43. 26389122 10.3389/fsurg.2015.00043 PMC4558465

[7] Gelhard, S., O’Brien, L., Vincenti, S., Smego, D. R., Hobbs, R., Varghese, T. K., Selzman, C. H., & Pereira, S. J. (2024). Disparities in gender and diversity representation among surgical subspecialties: Are we losing momentum? *Journal of Surgical Research,**293*, 413–419. 37812874 10.1016/j.jss.2023.08.051

[8] Moher, D., Liberati, A., Tetzlaff, J., Altman, D. G., PRISMA Group. (2010). Preferred reporting items for systematic reviews and meta-analyses: The PRISMA statement. *International Journal of Surgery,**8*(5), 336–341. 20171303 10.1016/j.ijsu.2010.02.007

[9] Sebo, P. (2021). Using genderize.io to infer the gender of first names: how to improve the accuracy of the inference. *Journal of the Medical Library Association,**109*(4), 609–612. 34858090 10.5195/jmla.2021.1252 PMC8608220

[10] Lattanza, L. L., Meszaros-Dearolf, L., O’Connor, M. I., Ladd, A., Bucha, A., Trauth-Nare, A., & Buckley, J. M. (2016). The Perry Initiative’s medical student outreach program recruits women into orthopaedic residency. *Clinical Orthopaedics and Related Research,**474*(9), 1962–1966. 27245771 10.1007/s11999-016-4908-y PMC4965379

[11] Hsu, M., Spurr, H., Cooper, A. P., & Schaeffer, E. K. (2025). The women in ORTHopaedics Program offers early exposure to orthopaedic surgery for young women. A pre- and post-event survey comparison. *JPOSNA,**10*, 100139. 40433576 10.1016/j.jposna.2024.100139 PMC12088246

[12] Jain, A. K. (2016). Current state of orthopedic education in India. *Indian Journal of Orthopaedics,**50*(4), 341–344. 27512213 10.4103/0019-5413.185586 PMC4964764

[13] Paul, D., Ghoshdastidar, S., Halder, S., & Sarkar, D. K. (2023). Are women finally joining orthopedics in India? A study of the causes limiting the number of women in orthopedics in India with steps for furthering progress. *Indian Journal of Orthopaedics,**57*(4), 586–595. 37006728 10.1007/s43465-023-00834-5 PMC10050455

[14] SICOT. 2023. Inspiring symphonies: Super seven of WOICE [Internet]. [place unknown]: SICOT Organization. http://www.sicot.org/wio2023. Reviewed 29 Mar 2025; Cited 7 May 2025.

[15] Asia Pacific Orthopaedic Association (APOA). 2023. Asia Pacific Women’s Advocacy [Internet]. Putrajaya, Malaysia: APOA. https://apoaonline.com/course/index.php?categoryid=27. Reviewed 29 Mar 2025; Cited 7 May 2025.

