# Supplementary material for: Gender Equity Issues in Orthopaedics: A Scoping Review
Source: Indian J Orthop. 2025 May 26;59(10):1609–20. doi: 10.1007/s43465-025-01415-4 (PMC12535563; doi:10.1007/s43465-025-01415-4)
Supplement: Supplementary file 1 — Supplementary file1 (DOCX 15 KB) [file 43465_2025_1415_MOESM1_ESM.docx]

**Supplemental Table S1. Search strategy for Global Index Medicus**

| **#** | **Terms** | **Rationale** |
| --- | --- | --- |
| 1 | (orthopedic$ or orthopaedic$ or orthopod$) | Orthopaedic-related keywords were added to capture as many papers on orthopaedics as possible. |
| 2 | AND | Step 2 was done to combine articles related to orthopaedics and gender equity in Step 1 and 3, respectively. |
| 3 | ((gender or women or woman or female or pregnancy) and (bias or diversity or stigma or norms or leadership or autonomy or train$ or training or competence or microaggression$ or burnout or dropout$ or equity or inequity or discrimination or equality or inequality or issue$ or barrier$ or inclusivity or inclusion or EDI or mentorship or representation or disparity or disparities or experience$ or challenge$ or compensation or authorship or flexibility or skill$ or pay or undervalue$ or opportunit$​)) | Gender equity-related keywords were added to capture as many papers on gender equity as possible. |
