# Supplementary material for: Gender Equity Issues in Orthopaedics: A Scoping Review
Source: Indian J Orthop. 2025 May 26;59(10):1609–20. doi: 10.1007/s43465-025-01415-4 (PMC12535563; doi:10.1007/s43465-025-01415-4)
Supplement: Supplementary file 2 — Supplementary file2 (DOCX 24 KB) [file 43465_2025_1415_MOESM2_ESM.docx]

**Supplemental Table S2. Study Distribution by Journal**

| **Journal** | **Gender Diversity & Representation** | **Research Productivity & Authorship** | **Leadership & Mentorship** | **Microaggressions & Lived Experiences** | **Monetary Aspects** | **Gender-based Health Impacts** | **Geographic Representation** | **Miscellaneous** | **TOTAL** |
| --- | --- | --- | --- | --- | --- | --- | --- | --- | --- |
| **Academic Medicine: Journal of the Association of American Medical Colleges** | 1 |  |  |  |  |  |  |  | **1** |
| **Acta Orthopaedica Belgica** | 1 |  |  |  |  |  |  |  | **1** |
| **American Journal of Orthopaedics (Belle Mead, N.J.)** |  | 1 |  |  |  |  |  |  | **1** |
| **American Journal of Surgery** |  |  |  | 1 |  |  |  |  | **1** |
| **ANZ Journal of Surgery** |  |  |  | 1 |  |  |  |  | **1** |
| **Arthroscopy, Sports Medicine, and Rehabilitation** | 1 |  | 2 |  |  |  |  |  | **3** |
| **Arthroscopy: The Journal of Arthroscopic & Related Surgery** | 1 |  |  |  |  |  |  |  | **1** |
| **Bone & Joint Open** | 2 |  |  |  |  | 1 |  | 1 | **4** |
| **Bone and Joint Journal** |  |  |  | 1 |  | 1 |  | 1 | **3** |
| **Canadian Journal of Surgery** | 1 |  | 1 | 1 |  |  |  |  | **3** |
| **Clinical Journal of Sport Medicine** | 1 |  |  |  |  |  |  |  | **1** |
| **Clinical Orthopaedics and Related Research** | 4 | 5 | 3 | 5 | 2 | 1 |  | 2 | **22** |
| **Cureus** | 2 | 3 | 2 | 1 |  |  | 1 |  | **9** |
| **Current Orthopaedic Practice** |  |  | 1 | 1 |  |  |  |  | **2** |
| **Foot & Ankle International** | 1 |  |  |  |  |  |  |  | **1** |
| **Foot & Ankle Orthopaedics** | 1 |  | 1 |  |  |  |  |  | **2** |
| **Foot & Ankle Specialist** | 1 |  |  |  |  |  |  |  | **1** |
| **Heliyon** |  |  |  |  |  |  |  | 1 | **1** |
| **HSS Journal: The Musculoskeletal Journal of Hospital for Special Surgery** | 1 | 1 | 1 |  |  |  |  |  | **3** |
| **Injury** | 1 |  |  |  |  |  |  |  | **1** |
| **JB & JS Open Access** | 5 | 3 | 2 | 1 | 1 | 1 |  | 1 | **14** |
| **JBJS Reviews** | 1 |  |  |  |  |  |  |  | **1** |
| **Journal of Bone and Joint Surgery** | 5 | 4 | 6 | 1 | 3 | 1 |  | 3 | **23** |
| **Journal of Graduate Medical Education** |  |  |  |  |  |  | 1 |  | **1** |
| **Journal of Healthcare Leadership** |  |  |  | 1 |  |  |  |  | **1** |
| **Journal of ISAKOS** |  |  | 1 |  |  |  |  |  | **1** |
| **Journal of ISAKOS: Joint Disorders & Orthopaedic Sports Medicine** |  |  | 1 |  |  |  |  |  | **1** |
| **Journal of Orthopaedic Research** |  | 1 |  |  | 1 |  |  |  | **2** |
| **Journal of Orthopaedic Science** |  | 1 |  |  |  |  |  |  | **1** |
| **Journal of Orthopaedic Trauma** |  | 1 | 1 |  |  |  |  |  | **2** |
| **Journal of Orthopaedics** |  | 1 |  |  | 1 | 1 |  |  | **3** |
| **Journal of Pediatric Orthopaedics** |  | 3 |  |  |  | 2 |  |  | **5** |
| **Journal of Shoulder and Elbow Surgery** | 2 | 1 | 2 | 1 |  |  |  |  | **6** |
| **Journal of Surgical Education** |  |  |  | 1 |  |  |  | 1 | **2** |
| **Journal of Taibah University Medical Sciences** |  | 1 |  |  |  |  |  |  | **1** |
| **Journal of the American Academy of Orthopaedic Surgeons. Global research & reviews** | 1 | 2 | 1 | 1 |  |  | 1 |  | **6** |
| **Malaysian Orthopaedic Journal** |  |  |  | 1 |  |  |  |  | **1** |
| **Military Medicine** | 1 |  |  |  |  |  |  |  | **1** |
| **Musculoskeletal Surgery** |  | 1 |  |  |  |  |  |  | **1** |
| **Occupational Medicine (Oxford, England)** |  |  |  |  |  | 1 |  |  | **1** |
| **Orthopaedic Journal of Sports Medicine** |  | 1 |  |  |  |  | 1 |  | **2** |
| **Orthopaedics & Traumatology, Surgery & Research: OTSR** |  | 1 |  |  |  |  |  |  | **1** |
| **Orthopedics** | 1 |  | 1 |  |  |  |  |  | **2** |
| **Revista Chilena de Ortopedia y Traumatologia** |  |  | 1 |  |  |  |  |  | **1** |
| **Spine** | 1 |  |  |  |  |  |  |  | **1** |
| **Surgery in Practice and Science** |  | 1 |  |  |  |  |  |  | **1** |
| **The Iowa Orthopaedic Journal** |  |  |  | 2 |  | 1 |  |  | **3** |
| **The Journal of Arthroplasty** | 3 | 2 |  | 2 |  |  |  |  | **7** |
| **The Journal of Knee Surgery** |  |  |  |  | 1 |  |  |  | **1** |
| **The Journal of the American Academy of Orthopaedic Surgeons** | 5 | 5 | 2 | 1 |  | 2 |  |  | **15** |
| **The New Zealand Medical Journal** |  |  |  |  |  |  |  | 1 | **1** |
| **The Orthopedic Clinics of North America** |  |  | 1 |  |  |  |  |  | **1** |
| **The Spine Journal: official journal of the North American Spine Society** |  | 1 |  |  |  |  |  |  | **1** |
| **The Surgeon: Journal of the Royal Colleges of Surgeons of Edinburgh and Ireland** |  |  |  |  | 1 |  |  |  | **1** |
| **Women’s Health Reports (New Rochelle, N.Y.)** | 1 |  |  |  |  |  |  |  | **1** |
| **World Journal of Orthopaedics** |  |  |  |  |  |  | 1 |  | **1** |
| **World Journal of Surgery** |  |  |  | 1 |  | 1 |  |  | **2** |
| **Zeitschrift fur Orthopadie und Unfallchirurgie** |  | 1 |  |  |  |  |  |  | **1** |
| **TOTAL** | **45** | **41** | **30** | **24** | **10** | **13** | **5** | **11** | **179** |
