# Supplementary material for: Gender Equity Issues in Orthopaedics: A Scoping Review
Source: Indian J Orthop. 2025 May 26;59(10):1609–20. doi: 10.1007/s43465-025-01415-4 (PMC12535563; doi:10.1007/s43465-025-01415-4)
Supplement: Supplementary file 3 — Supplementary file3 (DOCX 86 KB) [file 43465_2025_1415_MOESM3_ESM.docx]

**Table S3. Summary of Characteristics of Included Articles in the Gender Diversity & Representation Theme**

| **Title** | **Journal** | **Year** | **Author** | **First Author Gender** | **Corresponding**  **Author Gender** | **Country** | **Study type** | **Main Study Finding** |
| --- | --- | --- | --- | --- | --- | --- | --- | --- |
| **Residency** | | | | | | | | |
| Women in orthopedic surgery residencies in the United States | Academic medicine: journal of the Association of American Medical Colleges | 1998 | Biermann et al.^1^ | F | F | USA | Retrospective Review | There has been an increase in the percentage of women in orthopaedic residency, however, it has also been shown that women choose orthopaedics as a specialty less often than men. |
| A 5-Year Update on the Uneven Distribution of Women in Orthopaedic Surgery Residency Training Programs in the United States | Journal of Bone and Joint Surgery | 2016 | Van Heest et al.^2^ | F | F | USA | Retrospective review | Female medical students continue to pursue orthopaedic surgery as a career at rates lagging behind all other surgical specialties. |
| Current Trends in Sex, Race, and Ethnic Diversity in Orthopaedic Surgery Residency | The Journal of the American Academy of Orthopaedic Surgeons | 2019 | Poon et al.^3^ | F | F | USA | Retrospective Review | Although female representation in orthopaedics increased, the rate of increase was significantly lower compared with the majority of specialties. |
| Trends in Race and Sex Representation Among Entering Orthopaedic Surgery Residents: A Continued Call for Active Diversification Efforts | The Journal of the American Academy of Orthopaedic Surgeons | 2023 | Wang et al.^4^ | F | M | USA | Retrospective Review | Although there has been increased sex diversity within orthopaedic residency, other specialties have had a higher increase. |
| Gender trends in orthopedic surgical residency programs in Canada over 20 years | Gender trends in orthopedic surgical residency programs in Canada over 20 years | 2025 | Frazer et al.^5^ | F | F | Canada | Retrospective Review | While there is no gender effect in residency match rates, there are more male than female students that applied to orthopaedics, with no change of this trend over time. |
| Racial and Sex Disparities in Resident Attrition in Orthopaedic Surgery | JB & JS open access | 2023 | Haruno et al.^6^ | M | F | USA | Retrospective Review | Women residents had higher percentages of attrition and relative risk for experiencing attrition in comparison to men. |
| Orthopedic Surgery Residency Program Rankings and Gender Diversity | Cureus | 2024 | Han et al.^7^ | F | F | USA | Cross-sectional analysis | Higher ranked orthopaedic surgery residency programs were associated with greater gender diversity than lower ranked ones. |
| A 5-Year Update and Comparison of Factors Related to the Sex Diversity of Orthopaedic Residency Programs in the United States | JB & JS open access | 2023 | Julian et al.^8^ | F | F | USA | Retrospective Review | In the timespan of 5 years, the percentage of female orthopaedic residents and interns have increased, with there being higher percentages of female residents in programs with female faculty. |
| A 15-Year Report on the Uneven Distribution of Women in Orthopaedic Surgery Residency Training Programs in the United States | JB & JS open access | 2021 | Van Heest et al.^9^ | F | F | USA | Retrospective Review | Although the percentage of programs training women in orthopaedics residency have steadily increased, the majority of residency programs have no female trainees. |
| The uneven distribution of women in orthopaedic surgery resident training programs in the United States | The Journal of bone and joint surgery. American volume | 2012 | Van Heest et al.^10^ | F | F | USA | Retrospective Review | Orthopaedic residency programs in the USA don’t train women at an equal frequency as men, as the majority of programs have had no female trainees for one year or more |
| Correlations Between Department and Training Program Online Presence and Women in Orthopedic Surgery Training | Women's health reports (New Rochelle, N.Y.) | 2023 | Adkins et al.^11^ | F | F | USA | Retrospective Review | Digital media can be used to address the concerns in orthopaedics and to advocate for more gender diversity in the field |
| The Impact of Sustained Outreach Efforts on Gender Diversity in Orthopaedic Surgery | Journal of Bone and Joint Surgery | 2024 | Buckley et al.^12^ | F | F | USA | Modelling | Modelling estimates show incoming PGY1 class will continue to diversify until it reaches a peak of 28.0% ± 1.6% women in 2028. A steady state of 27.1% ± 1.3%, will hold until at least 2040. The field will reach a steady-state composition of approximately 25% women in practice by 2055. |
| **Fellowship** | | | | | | | | |
| Women in Orthopaedic Fellowships: What Is Their Match Rate, and What Specialties Do They Choose? | Clinical orthopaedics and related research | 2016 | Cannada et al.^13^ | F | F | USA | Retrospective Review | Female applicants for orthopaedic training in higher levels had a higher match rate than men in fellowship training, with the highest proportion of women in paediatric orthopaedics, and the lowest in spine. |
| Gender Diversity in Orthopaedic Surgery Residencies Does Not Translate to Accreditation Council for Graduate Medical Education-Accredited Fellowships | JB & JS open access | 2024 | Haddad et al.^14^ | M | M | USA | Retrospective Review | Even though the amount of female orthopaedic residents has increased in ACGME-accredited programs, this increase has not been shown in orthopaedic fellowship positions that are ACGME-accredited |
| Evaluation of Sex, Ethnic, and Racial Diversity Across US ACGME-Accredited Orthopedic Subspecialty Fellowship Programs. | Orthopedics | 2018 | Poon et al.^15^ | F | F | USA | Retrospective Review | Although there has been a slow increase in gender diversity in orthopaedic fellowship, the same increase has not been seen amongst orthopaedic residency as well as in racial and ethnic minorities. |
| Factors Associated With Increased Sex Diversity in Orthopaedic Trauma Association-Accredited Fellowship Training | The Journal of the American Academy of Orthopaedic Surgeons | 2024 | Silvestre et al.^16^ | M | M | USA | Cross-sectional analysis | There was an increase in the number of fellows, as well as female representation in orthopaedics between 2009 and 2024. |
| STEP 1: The Scottish Trauma & Orthopaedics Equality Project | Bone & joint open | 2025 | Jabbal et al.^17^ | M | F | Great Britain | Survey Study | Compared to other trauma and orthopaedic studies, the findings showed a higher percentage of female surgeons, with the highest female proportion in junior trainees. |
| Analysis of the diversity pipeline for the orthopedic trauma surgeon workforce in the United States | Injury | 2024 | Silvestre et al.^18^ | M | M | USA | Retrospective Review | Relative to medical school, orthopaedic surgery residency, and the US population, there are fewer female trainees in orthopaedic trauma fellowship training, with their being an overrepresentation of males in orthopaedic trauma. |
| Analysis of Sex Diversity Within Orthopedic Trauma Surgery Fellowship Programs | HSS journal: the musculoskeletal journal of Hospital for Special Surgery | 2024 | Cho et al.^19^ | F | F | USA | Cross-sectional analysis | Female surgeons are still underrepresented in orthopaedic trauma fellowship director roles, even though there has been an increase in the percentage of females entering orthopaedic trauma surgery fellowship programs. |
| Sex Diversity in the Emerging United States Arthroplasty Workforce Is Limited | The Journal of arthroplasty | 2024 | Silvestre et al.^20^ | M | M | USA | Retrospective Review | The percentage of women in arthroplasty fellowship has significantly increased between the years of 2012 and 2022 in comparison to the increase in fellows seen in both allopathic medicine and orthopaedic surgery residents. |
| Trends in Women Adult Reconstruction Surgeons: Fellowship Applicants and American Association of Hip and Knee Surgeons Members | The Journal of arthroplasty | 2025 | Xu et al.^21^ | F | F | USA | Retrospective Review | Although the percentage of women in the AAHKS and arthroplasty has increased over the time, the percentage compared to men in arthroplasty is relatively low. |
| Trends in Racial, Ethnic, and Gender Diversity in Orthopaedic Surgery Adult Reconstruction Fellowships From 2007 to 2021 | The Journal of arthroplasty | 2023 | Ajayi et al.^22^ | M | M | USA | Retrospective Review | There has been limited progress of increasing gender diversity in orthopaedics, specifically in adult reconstruction |
| Analysis of Gender Diversity Within Foot and Ankle Surgery Fellowship Programs | Foot & ankle international | 2024 | Cho et al.^23^ | F | M | USA | Cross-sectional analysis | The majority of programs with foot and ankle surgery faculty have no female representation, with the majority that do being programs in the Northeast of the USA, and the minority being in the Midwest of the USA. |
| A Comparison of Demographic Diversity Between Orthopaedic Surgery Residents and ACGME Foot and Ankle Fellows From 2007 to 2022 | Foot & ankle orthopaedics | 2024 | Joshi et al.^24^ | M | M | USA | Retrospective Review | While the majority of orthopaedic fellows are male, there has been an increase in the percentage of women in both orthopaedic fellowship as well as residency. |
| Persistent Lack of Female Orthopaedic Sports Medicine Fellows | Arthroscopy, sports medicine, and rehabilitation | 2023 | Lavorgna et al.^25^ | F | F | USA | Retrospective Review | The sports medicine field is still heavily male dominated, and the majority of sports medicine programs do not have a women’s sports medicine program in the USA. |
| Sex, Race, and Ethnic Diversity of the Emerging U.S. Orthopaedic Sports Medicine Workforce Is Limited | Arthroscopy: the journal of arthroscopic & related surgery: official publication of the Arthroscopy Association of North America and the International Arthroscopy Association | 2024 | Silvestre et al.^26^ | M | M | USA | Retrospective Review | Although the representation of women has increased in orthopaedics, the representation of female trainees has decreased from each stage of the training process, with females being underrepresented and males being overrepresented specifically in orthopaedic sports fellowship. |
| Trends in Racial, Ethnic, and Gender Diversity in Orthopedic Surgery Spine Fellowships From 2007 to 2021 | Spine | 2023 | Trenchfield et al.^27^ | M | M | USA | Retrospective Review | Orthopaedic spine surgery fellowship has not seen much progress in gender diversity, with the number of males being consistently high and the number of women being low. |
| **Faculty/Practice** | | | | | | | | |
| Female representation in orthopedic surgery: where do we stand in Belgium? | Acta orthopaedica Belgica | 2023 | Meert et al.^28^ | F | F | Belgium | Survey study + retrospective review | The findings of the survey demonstrated the need to address the underrepresentation of women in orthoapedic surgery. |
| How Does Orthopaedic Surgeon Gender Representation Vary by Career Stage, Regional Distribution, and Practice Size? A Large-Database Medicare Study | Clinical orthopaedics and related research | 2023 | Rodgers et al.^29^ | M | M | USA | Retrospective Review | There are substantially more men practicing orthopaedic surgery than women, with men having more years of practice than women. |
| Orthopaedic Surgery Faculty: An Evaluation of Gender and Racial Diversity Compared with Other Specialties | JB & JS open access | 2020 | Shah et al.^30^ | M | M | USA | Retrospective Review | While the representation of females in orthopaedic surgery and the URM faculty have increased over time, the increase is not as abundant as other specialties, with fewer female URM members having senior faculty status compared to other specialties. |
| Academic Gender Disparity in Orthopedic Surgery in Canadian Universities | Cureus | 2020 | Yue et al.^31^ | X | X | Canada | Cross-sectional analysis | Women are underrepresented in number, rank and academic productivity, with women holding consistently less leadership positions compared to their male counterparts |
| Academic Orthopaedics As a Driver of Gender Diversity in the Orthopaedic Workforce: A Review of 4,519 Orthopaedic Faculty Members | Journal of the American Academy of Orthopaedic Surgeons. Global research & reviews | 2022 | Kuhns et al.^32^ | M | M | USA | Cross-sectional analysis | Although there have been programs designed to increase gender diversity, the increase in orthopaedics is low in comparison to other surgical specialties. |
| How Long Will It Take to Reach Gender Parity in Orthopaedic Surgery in the United States? An Analysis of the National Provider Identifier Registry | Clinical orthopaedics and related research | 2021 | Acuna et al.^33^ | M | M | USA | Retrospective Review | With the current slow increase of women who are orthopaedic surgeons, it will take more than 200 years to reach gender parity within the specialty |
| Sex Diversity and Equity Among Fellows of the American Orthopaedic Association | The Journal of bone and joint surgery. American volume | 2025 | Silvestre et al.^34^ | M | M | USA | Retrospective Review | Despite being outnumbered significantly by male AOA members, women have a greater representation than expected among each academic rank compared to their proportional membership representation. |
| Trends in Gender Diversity Among Total Hip Arthroplasty Surgeons | The Journal of the American Academy of Orthopaedic Surgeons | 2024 | Oyem et al.^35^ | F | M | USA | Retrospective Review | Women arthroplasty surgeons billed significantly less procedures compared to males, with the males having a steady increase in the number of procedures performed and females having no increase or decrease. |
| Gender differences among shoulder arthroplasty surgeons: past, present, and future | Journal of shoulder and elbow surgery | 2024 | Wright et al.^36^ | F | M | USA | Retrospective Review | The number of arthroplasty shoulder surgeries performed by women has increased over time, however, men are still performing the majority of these surgeries |
| Differences in primary total shoulder arthroplasty volume, reimbursement, practice styles, and patient populations based on surgeon gender: a temporal analysis | Journal of shoulder and elbow surgery | 2024 | Gill et al.^37^ | M | M | USA | Retrospective Review | Female representation within the total shoulder arthroplasties has increased across the USA, with the greatest representation in the Northeast and West and the lowest representation in the South and Midwest. |
| Gender diversity in the National Joint Registry | Bone & joint open | 2024 | Lastoria et al.^38^ | M | M | Great Britain | Retrospective Review | There are very low percentages of contributing women surgeons in the National Joint Registry, with knee arthroplasty having the lowest female representation |
| Gender Disparities Among Professional Team Sports Medicine Physicians | Clinical journal of sport medicine: official journal of the Canadian Academy of Sport Medicine | 2023 | Schick et al.^39^ | M | M | USA | Retrospective Review | Compared with the present orthopaedic surgeons, there was less representation of female orthopaedic physicians at AOSSM and AAOS. |
| Diversity Within the Field of Orthopedic Sports Medicine: A Systematic Review | JBJS reviews | 2023 | Leal et al.^40^ | M | M | USA | Systematic review | The largest percentage of females was in paediatric orthopaedics and sports medicine had the third lowest |
| Race, Ethnicity, and Gender Representation Among US Academic Spine Surgeons | The Journal of the American Academy of Orthopaedic Surgeons | 2024 | Opara et al.^41^ | F | M | USA | Cross-sectional analysis | There is a significant gender disparity within the spine faculty, with there being no increase for minorities such as Black women and Hispanic/Latina women. |
| Gender and Geographic Trends Among Foot and Ankle Surgeons: Where Are We and Where Do We Need to Improve? | Foot & ankle specialist | 2024 | Shazadeh et al.^42^ | M | M | USA | Retrospective Review | While there has been as increase of females in orthopaedic foot and ankle surgeons, the numbers still remain low, with the most surgeons being in the south Atlantic, and the least amount in the East South Central. |
| **Overall** | | | | | | | | |
| Gender Disparities Within US Army Orthopedic Surgery: A Preliminary Report | Military medicine | 2018 | Daniels et al.^43^ | M | M | USA | Cross-sectional analysis | There are gender disparities amongst army orthoapedic surgeons and trainees, which is similar to that of civilian orthopaedics. |
| Diversity based on race, ethnicity, and sex between academic orthopaedic surgery and other specialties: a comparative study | The Journal of bone and joint surgery. American volume | 2010 | Day et al.^44^ | M | F | USA | Cross-sectional analysis | Orthopaedic surgery lags behind other surgical specialties in representing women. |
| Has diversity increased in orthopaedic residency programs since 1995? General | Clinical Orthopaedics and Related Research | 2012 | Daniels et al.^45^ | M | M | USA | Survey Study | When looking at orthopaedics and gender diversity overall, there has been an increase in the percentage of female orthopaedic surgeries, a smaller proportion of women in orthopaedic surgery fellowship, and a small percentage of women researchers. |

**Table S4. Summary of Characteristics of Included Articles in the Research & Authorship Theme**

| **Title** | **Journal** | **Year** | **Author** | **First Author Gender** | **Corresponding**  **Author Gender** | **Country** | **Study type** | **Main Study Finding** |
| --- | --- | --- | --- | --- | --- | --- | --- | --- |
| **Conference Involvement** | | | | | | | | |
| Representation of Female Speakers at the American Academy of Orthopaedic Surgeons Annual Meetings Over Time | The Journal of the American Academy of Orthopaedic Surgeons | 2023 | Nwosu et al.^46^ | F | F | USA | Retrospective Review | There has been increase in female speakers at AAOS meetings from 2009 to 2019, along with an increase in the percentage of female moderators and nontechnical sessions. |
| Gender disparities among speakers at major spine conferences | The spine journal: official journal of the North American Spine Society | 2023 | Lee et al.^47^ | F | M | USA | Retrospective Review | The percentage of women as invited speakers and moderators for academic conferences for six spine societies increased significantly over the study period, with annual increases of 8.8% and 20.8%, respectively, from 2013 to 2022. |
| Greater Gender Diversity Observed at Orthopaedic Conferences in the Caribbean Than in the United States or England | Cureus | 2022 | Mencia et al.^48^ | M | M | Trinidad & Tobago | Retrospective Review | Even though there was a steady increase in the number of presentations by women during the 5-year study period, with a mean value of 19.5%, there are significantly fewer presentations by women than men at the annual TCOS meetings. |
| Gender Representation in Speaking Roles at the American Association of Hip and Knee Surgeons Annual Meeting: 2012-2019 | The Journal of arthroplasty | 2021 | Cohen-Rosenblum et al.^49^ | F | F | USA | Retrospective Review | There were far fewer female presenters at AAHKS annual meetings |
| Abstracts accepted for the 2021-2023 French Orthopaedic and Traumatology Society meetings: Proportion of women submitters | Orthopaedics & traumatology, surgery & research: OTSR | 2024 | Klein et al.^50^ | F | F | France | Retrospective Review | A smaller proportion of women than men had abstracts accepted for presentation at the 2021, 2022, and 2023 SOFCOT meetings. |
| Gender Representation in Major Orthopaedic Surgery Meetings: A Quantitative Analysis | JB & JS open access | 2023 | Vivekanantha, et al.^51^ | M | M | Canada | Cross-sectional analysis | High prevalence of male-only panels (58.5%) and lack of female representation (12.6%) in 10 major Orthopaedic Surgery meetings despite similar qualifications academically. Conferences organized by the COA, ORS, and POSNA had higher percentages of female representation, while spine surgery and adult hip/knee reconstruction sessions had more than 70% male-only panels and fewer than 10% female members. |
| Gender of presenters at orthopaedic meetings reflects gender diversity of society membership | Journal of orthopaedics | 2020 | Tougas et al.^52^ | F | F | USA | Retrospective Review | Men continue to hold a higher proportion of more respected roles within orthopaedic academia as seen in the annual meeting programs for 2008 and 2017 from ten North American [orthopaedic](https://www.sciencedirect.com/topics/medicine-and-dentistry/orthopedics) societies. |
| Gender Parity in Academic Leadership Roles at AOSSM Annual Meetings | Orthopaedic journal of sports medicine | 2021 | Potter et al.^53^ | M | M | USA | Retrospective Review | Even though the percentage of female moderators had a small increase from 6.0% in 2015 to 8.6% in 2019, they represented a small portion of moderators and course instructors at the AOSSM Annual Meeting from 2015 to 2019. |
| Academic Engagement of Women as Orthopaedic Surgeons at the Annual Meetings of the Japanese Orthopaedic Association From 2012 to 2022 | Cureus | 2024 | Nagamine et al.^54^ | F | F | Japan | Retrospective Review | Although the proportion of women as members of the JOA has been increasing gradually and more women have been involved in JOA annual meetings, the proportion of women as presenters, invited speakers, symposiasts, and chairpersons of oral and poster presentations has generally been lower compared with the proportion of women as JOA members. |
| Are Women Proportionately Represented as Speakers at Orthopaedic Surgery Annual Meetings? A Cross-Sectional Analysis | Clinical orthopaedics and related research | 2020 | Gerull et al.^55^ | F | F | USA | Cross-sectional analysis | Women society members were proportionally represented at annual meetings, with a higher proportion of women representation in programs that had a diversity effort. |
| Diversity on the American Academy of Orthopaedic Surgeons National Meeting Podium: Changes Over Two Decades | The Journal of the American Academy of Orthopaedic Surgeons | 2024 | Samineni, Aneesh et al.^56^ | M | M | USA | Retrospective Review | Despite the fact that the female cohort has fewer publications on average, the percentage of women presenting instructional course lectures and American Academy of Orthopaedic Surgeons (AAOS) courses has increased over the past 2 decades and exceeded the percentage they represent in the AAOS by over 50%. |
| Representation of women and underrepresented minorities among grand rounds speakers in orthopaedic surgery | Surgery in practice and science | 2022 | Snow et al.^57^ | F | F | USA | Retrospective Review | There was substantially less representation of women in ortho who gave grand round sessions |
| **Publications/Authorship** | | | | | | | | |
| Despite Growing Number of Women Surgeons, Authorship Gender Disparity in Orthopaedic Literature Persists Over 30 Years | Clinical orthopaedics and related research | 2020 | Brown et al.^58^ | F | F | USA | Cross-sectional analysis | Although the content and topics within published orthopaedic articles remain the same between men and women, there are significantly lower percentages of women who were first authors. Although there is an increase in orthopaedic surgeons and residents who are women, the same increase has not increased for senior authors who are women. |
| How Well Represented Are Women Orthopaedic Surgeons and Residents on Major Orthopaedic Editorial Boards and Publications? | Clinical orthopaedics and related research | 2020 | Rynecki et al.^59^ | F | F | USA | Retrospective Review | Among authors in the JBJS, JAAOS, and CORR there were lower percentages of authors who were women. |
| What Are the Rates and Trends of Women Authors in Three High-impact Orthopaedic Journals from 2006-2017? | Clinical orthopaedics and related research | 2020 | Hiller et al.^60^ | F | M | USA | Retrospective Review | There has been an increase in the percentage of women first authors in articles published in three high-impact orthopaedic journals from 2006 to 2017. |
| Authorship trends in the Journal of Orthopaedic Research: A bibliometric analysis | Journal of orthopaedic research: official publication of the Orthopaedic Research Society | 2018 | Seetharam et al.^61^ | M | F | USA | bibliometric analysis | There was an approximate 27% increase for both female first and corresponding authors from 1983 to 2015 in Journal of Orthopaedic Research (*JOR*®) as evidenced by this study which shows considerable progress has been made over the past 30 years in closing the gender gap in academic medicine. |
| The orthopedic gender gap: trends in authorship and editorial board representation over the past 4 decades | American journal of orthopedics (Belle Mead, N.J.) | 2012 | Okike et al.^62^ | M | M | USA | Retrospective Review | Between 1970 and 2007, female representation as first authors, last authors and editorial board members increased significantly in 2 prominent general orthopedics journals (The Journal of Bone and Joint Surgery: American Volume (JBJS) and Clinical Orthopaedics and Related Research (CORR) but these rates of increase were lower than those observed in other fields of medicine. |
| The gender role in the publishing of Authorships in high-impact orthopedic journals | Musculoskeletal surgery | 2024 | Vitale et al.^63^ | F | F | Italy | Retrospective Review | From 2015-2020, the paper publishes in the journals of orthopedics and ap |
| Trend of female first authorship in Journal of Orthopaedic Science, the official journal of the Japanese orthopaedic association from 2001 to 2021: An observational study | Journal of orthopaedic science: official journal of the Japanese Orthopaedic Association | 2024 | Saka et al.^64^ | F | F | Japan | Retrospective Review | Female first authorship in the journal of orthopaedics (JOS) has been increasing. However, the proportion of female authors remains low. |
| Gender Trends in Authorship in 6 Major Orthopaedic Journals | The Journal of bone and joint surgery | 2024 | Grant et al.^65^ | F | F | USA | bibliometric analysis | There was an increase in female middle authorship over the decade-long study period but a low level of female participation in 6 orthopaedic journal publications overall. |
| Comparing the gender diversity and affiliation trends of the authors for two orthopaedics journals from the Arab world | Journal of Taibah University Medical Sciences | 2021 | Khalifa et al.^66^ | M | M | Egypt | Cross-sectional analysis | From the two journals: the Egyptian Orthopaedic Journal (EOJ) and the Journal of Musculoskeletal Surgery and Research (JMSR) there was a significantly larger number of contributions by female authors in JMSR (75, 14.2%) than EOJ (2, 0.3%). |
| Trends in Female Authorship in Orthopaedic Literature from 2002 to 2021: An Analysis of 168,451 Authors | The Journal of bone and joint surgery. American volume | 2023 | Ghattas et al.^67^ | F | F | USA | bibliometric analysis | There have been increasing publication rates of female authors in US orthopaedic journals. The proportion of female first authors was significantly greater than the proportion of female senior authors. |
| Female Authorship in the US Orthopaedics Literature: A Bibliometric Analysis of Trends | The Journal of the American Academy of Orthopaedic Surgeons | 2023 | Okewunmi et al.^68^ | M | M | USA | bibliometric analysis | There has been increasing female authorship from 2011 to 2020 for female first authors, but not female last authors or total authorship. |
| Bibliometric Analysis of Research Areas, Publication Hierarchy and Gender Authorship in German University Orthopaedic Surgery | Zeitschrift fur Orthopadie und Unfallchirurgie | 2023 | Preut et al.^69^ | F | M | Germany | bibliometric analysis | In the present study, among senior physicians with a management function, the share of publishing surgeons was 59.1% for women, but 85.5% for men. In contrast, in the group of senior physicians without management function female and male surgeons were almost equally represented (57.5% vs. 60.5%) |
| Evolution and Trends in Male Versus Female Authorship of Articles in Flagship Orthopaedic Journals From 1995 to 2020 | The Journal of the American Academy of Orthopaedic Surgeons | 2022 | Powell et al.^70^ | F | F | USA | Retrospective Review | Female authorship in prominent orthopaedic journals has increased markedly from 1995 to 2020 with inter-journal differences in senior author gender disparity. |
| Analysis of Author Gender in the Pediatric Orthopaedic Literature from 2011 to 2020 | Journal of pediatric orthopedics | 2021 | Prior et al.^71^ | F | M | USA | Retrospective Review | There is an increasing proportion of publications in the pediatric orthopaedic literature with female authors and female first authors from 2011 to 2020 in 3 major pediatric orthopaedic journals [*Journal of Children’s Orthopaedics* (*JCO*), *Journal of Pediatric Orthopaedics* (*JPO*), and *Journal of Pediatric Orthopaedics Part B* (*JPO-B*)]. |
| A Gender Gap in Publishing? Understanding the Glass Ceiling in Pediatric Orthopaedic Surgery | Journal of pediatric orthopedics | 2021 | Johnson et al.^72^ | M | M | USA | Retrospective Review | In pediatric orthopaedic surgery, abstracts authored by women are less likely to reach publication, despite no identifiable differences in study quality |
| Male Versus Female Authorship in Flagship Pediatric Orthopaedic Journals From 2002 to 2021 | Journal of pediatric orthopedics | 2023 | Videckis et al.^73^ | M | M | USA | Retrospective Review | While female first authorship in prominent pediatric orthopaedic journals has increased significantly from 2002 to 2021, senior authorship has remained stagnant. |
| Disparities Among Leading Publishers of Arthroplasty Research | The Journal of arthroplasty | 2021 | Xu et al.^74^ | F | F | USA | Retrospective Review | In arthroplasty, there is a much smaller percentage of women who make up unique authors, and an even smaller percentage of women who are first authors |
| Trends in Gender Disparities in Authorship of Arthroplasty Research | The Journal of bone and joint surgery. American volume | 2020 | Xu et al.^75^ | F | F | USA | Retrospective Review | Of published arthroplasty orthopaedic articles, there were less women who were authors, with the majority of women being middle authors, and the majority of men being first authors |
| A 15-Year Bibliometric Analysis of Sports Medicine Studies in The Journal of Bone and Joint Surgery: A Systematic Review | JB & JS open access | 2024 | Watters et al.^76^ | F | F | USA | Retrospective Review | The gender disparity in authorship has remained stagnant with female authorship only increasing from 4.8 to 6.3% and females being only 11% of all first authors and 9% of senior authors in The Journal of Bone and Joint Surgery-American Volume (JBJS-A) from 2007 to 2021. |
| A 46-year Analysis of Gender Trends in Academic Authorship in Orthopaedic Sports Medicine | The Journal of the American Academy of Orthopaedic Surgeons | 2019 | Kim et al.^77^ | F | M | USA | Retrospective Review | Female authors have had a substantial increase from 1972 to 2018. With this being said, the top female authors are 3.4 less likely to be published than their male counterparts |
| Comparative Analysis of Bibliometric, Authorship, and Collaboration Trends Over the Past 30-Year Publication History of the Journal of Orthopaedic Trauma and Injury | Journal of orthopaedic trauma | 2018 | Russell et al.^78^ | F | M | USA | bibliometric analysis | Female first authorship in the *Journal of Orthopaedic Trauma* (*JOT*) is climbing at a higher rate (2.3 times) than the *Injury* Journal (1.5 times). |
| Research Productivity and Impact in Foot and Ankle Surgery: Insights From Relative Citation Ratio Analysis of Recent Fellowship Graduates | Journal of the American Academy of Orthopaedic Surgeons. Global research & reviews | 2024 | Benes et al.^79^ | M | M | USA | Retrospective Review | 674/820 fellows from the AOFAS database (2008/2009 to 2022/2023 academic years) were men. |
| A Bibliometric Analysis of the 500 Most Cited Papers in Orthopaedic Oncology | Journal of the American Academy of Orthopaedic Surgeons. Global research & reviews | 2024 | Fanfan et al.^80^ | M | M | USA | Retrospective Review | Female participation as first authors significantly increased from the 1960s to the 2010s (0% vs 14.6% as seen in the analysis of 500 most cited papers in orthopaedic oncology. |
| Gender disparities in shoulder and elbow publications | Journal of shoulder and elbow surgery | 2022 | Sequeira et al.^81^ | M | M | USA | Retrospective Review | Although female representation in first, senior, and general authorship lags behind male representation in the shoulder and elbow literature, female authorship has significantly increased since 2002. |
| Is There Gender Disparity in Orthopedic Surgery Resident Research Productivity? | HSS journal: the musculoskeletal journal of Hospital for Special Surgery | 2024 | Ellsworth et al.^82^ | F | F | USA | Cross-sectional analysis | Among all residents, male residents had more total publications, first or last author publications, and middle author publications than female residents from the top 10 ranked US orthopedic surgery residencies by research output in 2021–2022. |
| Gender Disparity in Authorship Among Orthopaedic Surgery Residents | JB & JS open access | 2024 | Cho et al.^83^ | F | F | USA | Cross-sectional analysis | Despite similar rates of first author publication among male and female residents, female residents had fewer publications overall, lower H-indices, and disproportionately fewer first author publications than would be expected given their representation. |
| **Clinical Trials/Research Groups** | | | | | | | | |
| Women Are Underrepresented Among Principal Investigators of Hip and Knee Arthroplasty Clinical Trials in the United States | The Journal of bone and joint surgery. American volume | 2023 | Silvestre et al.^84^ | M | M | USA | Cross-sectional analysis | Women were underrepresented among PIs of hip and knee arthroplasty clinical trials, which may lead to disparities in academic promotion and advancement. A total of 157 clinical trials involving 192 arthroplasty PIs were included of which only **2 (1.0%) were women.** |
| What Are the Trends in Women's Representation Among Lead Investigators of Orthopaedic Clinical Trials? | Clinical orthopaedics and related research | 2024 | Burkhart et al.^85^ | M | M | USA | Cross-sectional analysis | Although there has been a notable increase in the proportion of women principal investigators over time, the overall representation remains relatively low. |
| Orthopaedic Research Consortiums: A Review of Scope, Sex and Racial Representation | Cureus | 2024 | Ozdag et al.^86^ | M | M | USA | Cross-sectional analysis | While women in orthopaedic research consortiums remain under-represented relative to the proportion of women in medical school, their representation in research consortiums exceeds their representation in almost every orthopaedic professional society. |

**Table S5. Summary of Characteristics of Included Articles in the Leadership & Mentorship Theme**

| **Title** | **Journal** | **Year** | **Author** | **First Author Gender** | **Corresponding**  **Author Gender** | **Country** | **Study type** | **Main Study Finding** |
| --- | --- | --- | --- | --- | --- | --- | --- | --- |
| **Mentorship Influences** | | | | | | | | |
| Representation Matters: A Higher Percentage of Women Orthopaedic Surgery Faculty Is Associated With an Increased Number of Women Residents | The Journal of the American Academy of Orthopaedic Surgeons | 2024 | Ranson et al.^87^ | F | F | USA | Retrospective Review | A positive correlation (r = 0.577, α < 0.001) was found between the number of female faculty members and female residents in the residency programs, particularly those with more women in leadership positions. |
| Analysis of Factors Related to the Sex Diversity of Orthopaedic Residency Programs in the United States | The Journal of bone and joint surgery. American volume | 2018 | Sobel et al.^88^ | M | M | USA | Retrospective Review | Greater percentages of female residents were found at orthopaedic residency programs with more female faculty members, more women in leadership positions, a women’s sports medicine program, and the option to do a research year. |
| Residents' perceptions of sex diversity in orthopaedic surgery | The Journal of bone and joint surgery. American volume | 2013 | Hill et al.^89^ | F | F | USA | Survey Study | Increased exposure to female mentorship may help to recruit more women into the orthopaedic surgery workforce. |
| Effective Mentorship of Women and Underrepresented Minorities in Orthopaedic Surgery: A Mixed-Methods Investigation | JB & JS open access | 2022 | Winfrey et al.^90^ | F | F | USA | Survey Study | Mentorship was highly valued among women in orthopaedic surgery across all career stages, with medical students being the most likely to consider gender concordance with their mentor as important. |
| Personal Satisfaction and Perception of Success among Female Orthopedic Surgeons: Key Factors in the Choice of Specialty and Professional Development | Revista Chilena de Ortopedia y Traumatologia | 2022 | Ramirez et al.^91^ | F | F | Chile | Survey Study | The role played by teachers and mentors, as well as an interest in surgery and sports are very relevant when choosing orthopaedic surgery as a speciality. The majority of female respondents (58%) reported having felt greater difficulties than their male colleagues, and 73% considered that being a woman reduced their chances of success in the field. |
| Is Program Director Gender Associated With Gender Diversity Among Orthopaedic Surgery Residency Programs? | Clinical orthopaedics and related research | 2024 | Dias et al.^92^ | F | F | USA | Cross-sectional Analysis | Orthopaedic residency programs that were run by women did not contain a higher percentage of women residents. |
| A profile of female academic orthopaedic surgeons | Current Orthopaedic Practice | 2013 | Hill et al.^93^ | F | F | USA | Survey Study | Only 44% of female faculty members reported that they were positively influenced by mentors when they were junior faculty. Less than 23% of the faculty members surveyed indicated that a female mentor positively influenced them at each of these levels of training. |
| **Faculty Leadership Positions** | | | | | | | | |
| Sex, Race, and Ethnicity of Faculty and Department Chairs in Orthopaedic Surgery and Comparable Fields: 2015 to 2022 | The Journal of the American Academy of Orthopaedic Surgeons | 2024 | Budin et al.^94^ | M | F | USA | Retrospective Review | The representation of females in department chair positions within orthopaedics lags behind other specialties, as well as the field of medicine as a whole. |
| Diversity among academic orthopedic shoulder and elbow surgery faculty in the United States | Journal of shoulder and elbow surgery | 2020 | Chen et al.^95^ | M | M | USA | Cross-sectional analysis | There exists a lack of gender diversity within shoulder and elbow surgery, however gender did not have a significant effect on leadership rank or practice setting. |
| Gender Disparities in Endowed Professorships Within Orthopaedic Surgery | Cureus | 2024 | Asturias et al.^96^ | F | F | USA | Retrospective Review | Gender inequities at the endowment level are substantial, and there are very few women in musculoskeletal medicine to achieve endowed professorships. |
| Orthopaedic Sports Medicine Fellowship Directors Are Predominantly White Men With a High Degree of Research Productivity | Arthroscopy, sports medicine, and rehabilitation | 2021 | Moore et al.^97^ | M | M | USA | Retrospective Review | There is very little female representation amongst fellowship directors in sports medicine. |
| Orthopedic Surgery Fellowship Directors: Trends in Demographics, Education, Employment, and Institutional Familiarity | HSS journal: the musculoskeletal journal of Hospital for Special Surgery | 2023 | Kamalapathy et al.^98^ | M | M | USA | Retrospective Review | Gender demographics revealed that with respect to orthopaedic subspecialties, oncology, pediatrics, and hand had the highest percentage of Female fellowship directors. Of the 537 fellowship directors, 30 (5.6%) were women. |
| Demographics and Characteristics of Orthopaedic Surgery Residency Program Directors: A Cross-sectional Review | JB & JS open access | 2023 | Cummings et al.^99^ | F | M | USA | Retrospective Review | There is a sparsity of female program directors amongst orthopaedic residency programs. |
| Racial, Ethnic, and Gender Diversity in Academic Orthopaedic Surgery Leadership | The Journal of bone and joint surgery. American volume | 2022 | Meadows et al.^100^ | M | M | USA | Retrospective Review | There has been improvement in gender diversity among chairpersons in orthopaedic surgery leadership, however there was a significant decrease in gender representation observed between the 2019 orthopaedic faculty and the 2019/2020 orthopaedic leadership. |
| Gender disparity in academic orthopedic programs in Canada: a cross-sectional study | Canadian journal of surgery. Journal canadien de chirurgie | 2022 | Hunter et al.^101^ | F | F | Canada | Retrospective Review | In 2018/19, women orthopedic surgeons were underrepresented in faculty positions across academic orthopedic training programs in Canada, and were disproportionately underrepresented in promoted academic faculty roles and leadership positions. |
| Representation of Women in Academic Orthopaedic Leadership: Where Are We Now? | Clinical orthopaedics and related research | 2022 | Bi et al.^102^ | M | M | USA | Retrospective Review | The higher percentage of women in junior leadership positions in orthopaedic surgery, with the data available, is a promising finding. However, more improvement is needed to achieve gender parity in orthopaedics overall, and more information is needed in terms of publicly available information on gender representation in orthopaedic leadership. |
| Demographic and academic characteristics of orthopaedic shoulder and elbow division chiefs in the United States | Journal of shoulder and elbow surgery | 2025 | Proal et al.^103^ | M | M | USA | Cross-sectional Analysis | There is a scarcity of available research on demographic and leadership characteristics in academic orthopaedic shoulder and elbow, however current numbers show that only 8.2% of Division Chiefs are female. |
| Sex Disparities Among Fellowship Program Directors in Orthopaedic Surgery | The Journal of bone and joint surgery. American volume | 2024 | Silvestre et al.^104^ | M | M | USA | Retrospective Review | Women are underrepresented among orthopaedic fellowship Program Directors, especially in certain subspecialties (orthopaedic sports medicine, shoulder and elbow, and adult reconstruction). |
| **Society Leadership Positions** | | | | | | | | |
| A Five-year Review of the Designated Leadership Positions of Pediatric Orthopaedic Society of North America: Where Do Women Stand? | The Orthopedic clinics of North America | 2019 | Poon et al.^105^ | F | F | USA | Retrospective Review | The representation of women in leadership positions within POSNA is lower than the membership percentage. |
| Dissecting disparity: Improvements towards gender parity in leadership and on the podium within the Canadian Orthopaedic Association | Journal of ISAKOS | 2019 | Hiemstra et al.^106^ | F | F | Canada | Retrospective Review | While gender parity has yet to be achieved in Canadian orthopaedics, the proportion of females in leadership roles and on the podium is consistent with the current gender diversity within the Canadian Orthopaedic Association (COA) membership. |
| A 10-Year Review of Designated Leadership Positions of the American Orthopaedic Foot & Ankle Society (AOFAS) | Foot & ankle orthopaedics | 2022 | Chrea et al.^107^ | F | F | USA | Retrospective Review | The female membership of the American Orthopaedic Foot and Ankle Society (AOFAS) has similar gender composition to other orthopaedic subspecialties |
| Gender Diversity, Leadership, Promotion, and Opportunity Among the Members of the Orthopaedic Trauma Association (OTA) | Journal of orthopaedic trauma | 2023 | Murphy et al.^108^ | F | F | Canada | Retrospective Review | Gender disparity exists within the upper ranks of leadership and academic representation in the Orthopaedic Trauma Association (OTA). |
| Orthopaedic Society Leadership Diversity and Academic Participation: Where Do We Stand Now? | The Journal of bone and joint surgery. American volume | 2022 | Albright et al.^109^ | M | M | USA | Survey Study + Retrospective Review | There is a lack of sex diversity in orthopaedic society leadership. |
| Women in Leadership in State and Regional Orthopaedic Societies | Journal of the American Academy of Orthopaedic Surgeons. Global research & reviews | 2022 | Ramos et al.^110^ | F | F | USA | Survey Study | There is a positive correlation between female members and women on the Board of Directors in regional and state orthopaedic societies. |
| Does the Proportion of Women in Orthopaedic Leadership Roles Reflect the Gender Composition of Specialty Societies? | Clinical orthopaedics and related research | 2020 | Saxena et al.^111^ | F | F | USA | Survey Study | There is a correlation between the number of female members in an orthopaedic specialty society and the number of women on its board of directors. |
| Women in leadership in orthopaedic sports medicine societies throughout the world | Journal of ISAKOS: joint disorders & orthopaedic sports medicine | 2024 | Tanguilig et al.^112^ | F | F | Canada | Cross-sectional Analysis | While some countries have higher representation than others, the number of women in leadership positions in orthopaedic sports medicine societies throughout the world is significantly less than their male counterparts. |
| Representation of Male and Female Orthopedic Surgeons in Specialty Societies | Orthopedics | 2021 | Attia et al.^113^ | F | F | USA | Survey Study | Most orthopedic specialty societies have lower female than male membership, fewer women in leadership positions, and fewer monetary awards granted to women compared with men. This disparity was more evident in specialty societies than general orthopedic societies. |
| Diversity in Orthopaedic Sports Medicine Societies | Arthroscopy, sports medicine, and rehabilitation | 2023 | Steele et al.^114^ | F | F | USA | Retrospective Review | Although there has been an increase in representation of female orthopaedic surgeons within both the Arthroscopy Association of North America (AANA) and the American Orthopaedic Society of Sports Medicine (AOSSM), there is still room for more diversity and inclusion within committee membership and leadership. |
| **Other Leadership Positions** | | | | | | | | |
| Racial/Ethnic and Gender Diversity of Orthopaedic Journal Editorial Boards | The Journal of bone and joint surgery. American volume | 2024 | Pujari et al.^115^ | M | M | USA | Cross-sectional Analysis | The representation of women on the editorial boards included in this analysis was similar to their representation in academic orthopaedics, however, this value remains low in comparison with the population of patients treated by orthopaedic surgeons. |
| Orthopedic Team Surgeons in Major Professional Sports: An Analysis of Affiliation With the Top 10 Sports Medicine Fellowship Programs and Implications for Leadership and Diversity | Cureus | 2024 | Wood et al.^116^ | M | M | USA | Retrospective Review | Out of the surgeons listed as team surgeons in all of the major professional sports, female surgeons only represent 1.6% of the total. |

**Table S6. Summary of Characteristics of Included Articles in the Microaggressions & Lived Experiences Theme**

| **Title** | **Journal** | **Year** | **Author** | **First Author Gender** | **Corresponding**  **Author Gender** | **Country** | **Study type** | **Main Study Finding** |
| --- | --- | --- | --- | --- | --- | --- | --- | --- |
| **Microaggressions & Gender Bias** | | | | | | | | |
| The prevalence and impact of gender bias and sexual discrimination in orthopaedics, and mitigating strategies | The bone & joint journal | 2020 | Halim et al.^117^ | M | M | Great Britain | Systematic Review | Gender bias and sexual discrimination is common in the orthopaedic workplace, impacting representation, salaries and career success for women. |
| Gender-Related Microaggressions in Orthopedic Surgery: A Comprehensive Survey of Women Orthopedists and Implications for Progress, Saudi Arabia | Journal of healthcare leadership | 2024 | Alhammadi et al.^118^ | F | M | Saudi Arabia | Survey Study | Over 60% of women orthopaedic surgeons in Saudi Arabia reported being the victim of microaggression(s), with male orthopaedic surgeons and patients/families the most common perpetrators. |
| Perceptions of Racial and Gender Microaggressions in an Academic Orthopaedic Department | JB & JS open access | 2023 | Carino et al.^119^ | F | F | USA | Survey Study | Women members of an institution's ortho department experienced significantly more gender-based microaggressions than their men colleagues. |
| Interpersonal Interactions and Biases in Orthopaedic Surgery Residency: Do Experiences Differ Based on Gender? | Clinical orthopaedics and related research | 2023 | Sobel et al.^120^ | M | M | USA | Survey Study | Women orthopaedic residents reported experiencing microaggressions more frequently than men residents (40% vs 5%) in professional interactions, and women residents are more likely to experience sexual harassment and disparaging humour than men residents. |
| What Proportion of Women Orthopaedic Surgeons Report Having Been Sexually Harassed During Residency Training? A Survey Study | Clinical orthopaedics and related research | 2020 | Whicker et al.^121^ | F | F | USA | Survey Study | 68% of women reported experiencing sexual harassment during orthopaedic training, and this does not appear to have improved over time. |
| Prevalence of gender-based and sexual harassment within orthopedic surgery in Canada | Canadian journal of surgery. Journal canadien de chirurgie | 2022 | Giglio et al.^122^ | F | F | Canada | Survey Study | The prevalence of gender-based and sexual harassment is high within Canadian orthopedic surgery, and women are at highest risk for experiencing harassment (OR 16.2). |
| Do women experience microaggressions in orthopaedic surgery? Current state and future directions from a survey of women orthopaedists | Current Orthopaedic Practice | 2020 | Samora et al.^123^ | F | F | USA | Survey Study | Microaggressions are commonly experienced by women in orthopaedics (92.4%). The most common perpetrators of microaggressions are patients and their families, but other medical and support staff contribute to the environment. |
| Women in Orthopedics and their Fellowship Choice: What Influenced their Specialty Choice? | The Iowa orthopaedic journal | 2020 | Jurenovich et al.^124^ | F | F | USA | Survey Study | 79% of women were not influenced by a female role model in their career choice, and 18% of women felt discriminated against during the interview or fellowship process. |
| A Cross-Sectional Study of Gender-Specific Influences of Orthopedic Subspecialty Selection | The Iowa Orthopaedic Journal | 2024 | Koschmeder et al.^125^ | F | F | USA | Survey Study | Women trainees rated discrimination based on gender significantly higher than men, and placed greater import on choosing a subspecialty with faculty trainees of the same gender. |
| An investigation into gender bias in the evaluation of orthopedic trainee arthroscopic skills | Journal of shoulder and elbow surgery | 2022 | Leape et al.^126^ | F | M | USA | Survey Study | Trainee gender did not influence evaluator ratings and comments given for arthroscopic skills. |
| Female Residents Give Themselves Lower Scores Than Male Colleagues and Faculty Evaluators on ACGME Milestones | Journal of surgical education | 2021 | Brady et al.^127^ | F | F | USA | Retrospective Review | Women residents are at risk for a competency bias during training, as reflected by scoring themselves lower than men counterparts and faculty mentors on the ACGME Milestones. |
| The Effect of an Orthopaedic Surgeon's Attire on Patient Perceptions of Surgeon Traits and Identity: A Cross-Sectional Survey | Journal of the American Academy of Orthopaedic Surgeons. Global research & reviews | 2020 | Goldstein et al.^128^ | F | F | USA | Survey Study | Women surgeons who wear feminine business attire instead of scrubs may be perceived less able to perform the physical work of operating, but are otherwise rated comparably with their peers, both male and female. |
| Chair Versus Chairman: Does Orthopaedics Use the Gendered Term More Than Other Specialties? | Clinical orthopaedics and related research | 2020 | Peck et al.^129^ | M | F | USA | Retrospective Review | 60% of orthopaedic department websites used the gendered term "Chairman", suggesting there is considerable room for growth in the use of gender-equal language in orthopaedics. |
| **Barriers and Facilitators** | | | | | | | | |
| Barriers and facilitators for female practitioners in orthopaedic training and practice: a scoping review | ANZ journal of surgery | 2025 | Freeman et al.^130^ | F | F | Australia | Scoping Review | Most barriers for women orthopaedic surgeons existed at the organizational level and included male-dominated culture, gender discrimination and stereotypes, and workplace violence. |
| Motivations and Barriers for Women Orthopaedic Surgeons Considering Arthroplasty Fellowship | The Journal of arthroplasty | 2024 | Lancaster et al.^131^ | F | F | USA | Survey Study | Women arthroplasty surgeons cited concerns about work-life balance, pregnancy, and sex bias from referring physicians, while women who did not choose arthroplasty cited concerns about culture, physicality and lack of mentorship. |
| The Importance of Perceived Barriers to Women Entering and Advancing in Orthopaedic Surgery in the US and Beyond | World journal of surgery | 2023 | Xu et al.^132^ | F | F | USA | Survey Study | Male-dominated culture, lack of women mentors were barriers to entering orthopaedic surgery, while lack of women in leadership, family responsibilities, gender bias in promotions were barriers to advancement |
| Women in Orthopaedics: A Perspective from Malaysian Female Orthopaedic Surgeons | Malaysian orthopaedic journal | 2023 | Liew et al.^133^ | F | F | Malaysia | Survey Study | Reported barriers for Malaysian women orthopaedic surgeons include: physical strength required, gender bias and discrimination, verbal and physical harassment. |
| Factors That Influence Orthopedic Women Residents' Selection of Adult Reconstruction | The Journal of arthroplasty | 2023 | Lieberman et al.^134^ | F | F | USA | Survey Study | Women and men residents expressed similar rates of interest and self-confidence in Adult Reconstruction, but there were social barriers including negative stereotypes that may prevent women from pursuing careers in Adult Reconstruction. |
| Experiences of Canadian Female Orthopaedic Surgeons in the Workplace: Defining the Barriers to Gender Equity | The Journal of bone and joint surgery | 2022 | Hiemstra et al.^135^ | F | F | Canada | Survey Study | 5 barriers to workplace equity for Canadian female orthopaedic surgeons were identified by the Gender Bias Scale: Constrained Communication, Unequal Standards, Male Culture, Lack of Mentoring, Workplace Harassment. |
| Where Are the Women in Orthopaedic Surgery? | Clinical Orthopaedics and Related Research | 2016 | Rohde et al.^136^ | F | F | USA | Survey Study | Most women practicing orthopaedics were attracted to the field because of their individual personal affinity despite the lack of role models and exposure; improving mentorship and increasing early exposure may increase personal interest in the field. |
| **Lived Experiences** | | | | | | | | |
| Do Women and Minority Orthopaedic Residents Report Experiencing Worse Well-being and More Mistreatment Than Their Peers? | Clinical orthopaedics and related research | 2024 | Gerull et al.^137^ | F | F | USA | Survey Study | Women orthopaedic surgeons report experiencing more emotional exhaustion and more mistreatment than their peers. Women have more thoughts of leaving residency than men, which may contribute to attrition during training. |
| The Perspective of Brazilian Women Orthopaedic Surgeons on Gender Discrimination: Initial Insights to Understand Gender Bias in the Brazilian Healthcare System | Cureus | 2024 | Amaral et al.^138^ | F | M | Brazil | Survey Study | Women orthopaedic surgeons in Brazil more workplace conflicts and trainees expressed a feeling of inequality towards women in the workplace, which has physical and psychological consequences. |
| The other side of conflict: Examining the challenges of female orthopaedic surgeons in the workplace | American journal of surgery | 2023 | Rodarte et al.^139^ | F | F | USA | Survey Study | 72% of women orthopaedic surgeons reported experiencing gender-based workplace conflict, and 8% reported being forced out or leaving, leading to depression, anxiety and burnout. |
| **Workplace Violence** | | | | | | | | |
| Workplace Violence in Orthopaedic Surgery: A Survey of Academy of Orthopaedic Surgeons Membership | The Journal of the American Academy of Orthopaedic Surgeons | 2024 | Ponce et al.^140^ | M | M | USA | Survey Study | Women orthopaedic surgeons report more workplace violence events per practice year than men (2.25 vs 0.65) and increased likelihood of physical threats and assaults from co-workers. |

**Table S7. Summary of Characteristics of Included Articles in the Gender-based Health Impacts Theme**

| **Title** | **Journal** | **Year** | **Author** | **First Author Gender** | **Corresponding**  **Author Gender** | **Country** | **Study type** | **Main Study Finding** |
| --- | --- | --- | --- | --- | --- | --- | --- | --- |
| **Pregnancy and Fertility** | | | | | | | | |
| Pregnancy and infertility in orthopedics: A review of the current state | World Journal of Surgery | 2024 | Kermanshahi et al.^141^ | F | F | USA | Systematic review | Female orthopaedic trainees and attendings delay childbearing, experience higher rates of obstetric complications, and more stigma associated with pregnancy compared to their male colleagues. |
| UK pregnancy in orthopaedics (UK-POP): a cross-sectional study of UK female trauma and orthopaedic surgeons and their experiences of pregnancy | Bone & Joint Open | 2023 | Kontoghiorghe et al.^142^ | F | F | Great Britain | Survey study | Despite a large proportion of female trauma and orthopaedic (T&O) surgeons having and wanting children, T&O surgeons in the UK delay childbearing, experience bias, and also have high rates of infertility and obstetric complications. |
| What Are the Challenges Related to Family Planning, Pregnancy, and Parenthood Faced by Women in Orthopaedic Surgery? A Systematic Review | Clinical Orthopaedics and Related Research | 2023 | Morrison et al.^143^ | F | F | Canada | Systematic review | Orthopaedic surgeons are more likely to experience pregnancy complications, and many women delay childbearing. |
| Identifying Barriers: Current Breastfeeding Policy in Orthopedic Surgery Residency | The Iowa Orthopaedic Journal | 2021 | Wynn, Malynda et al.^144^ | F | F | USA | Retrospective review | Scarce information is available to prospective ortho residents regarding breastfeeding policies and lactation facilities, with only 2.8% of current programs having website information discussing breastfeeding support. |
| Does a Career in Orthopaedic Surgery Affect a Woman's Fertility? | The Journal of the American Academy of Orthopaedic Surgeons | 2021 | Poon et al.^145^ | F | F | USA | Survey study | Most women in orthopaedics desire children but delay childbearing due to their career choice and its demands. |
| The Perception of Pregnancy and Parenthood Among Female Orthopaedic Surgery Residents | The Journal of the American Academy of Orthopaedic Surgeons | 2019 | Mulcahey et al.^146^ | F | F | USA | Survey study | Most female orthopaedic trainees do not have children during residency, and many women experienced bias from co-residents and/or attendings about childbearing. |
| Pregnancy in Orthopaedic Residents: Peripartum Barriers Identified | JB & JS Open Access | 2022 | Ruse et al.^147^ | F | F | USA | Survey study | The 3 most prominent barriers to childbearing during residency were concerns about the ability to balance clinical and maternal duties, fear of judgment by those in the program, and an inability to ensure optimal prenatal and postpartum care. |
| Pregnancy, parenthood, and fertility in the orthopaedic surgeon | The Bone & Joint Journal | 2023 | Morgan et al.^148^ | F | F | Great Britain | Systematic review | Female orthopaedic surgeons have high rates of obstetric complications and infertility. |
| Fertility and pregnancy complications in female orthopaedic surgeons | Occupational Medicine (Oxford, England) | 2024 | Frenkel et al.^149^ | X | X | Israel | Survey study | Orthopaedic surgeons in Israel experience a delay in childbirth and higher rates of pregnancy complications. |
| **Burnout** | | | | | | | | |
| Rates of Burnout in Female Orthopaedic Surgeons Correlate with Barriers to Gender Equity | The Journal of Bone and Joint Surgery. American volume | 2023 | Hiemstra et al.^150^ | F | F | Canada | Survey study | 50.5% of the surveyed female orthopaedic surgeons experienced career burnout, which was significantly negatively correlated with age and job satisfaction. |
| Pediatric Orthopaedists Are Not Immune: Characterizing Self-reported Burnout Rates Among POSNA Members | Journal of Pediatric Orthopedics | 2020 | Carter et al.^151^ | F | F | USA | Survey study | Women were more likely to report both personal and team burnout than men. |
| An overview of occupational injuries among female orthopaedic surgeons | Journal of Orthopaedics | 2024 | Sedani et al.^152^ | M | M | USA | Survey study | Despite no difference in the rate of musculoskeletal injuries between males and females, women reported significantly higher rates of burnout, social isolation, and a need to seek counselling. |
| **Occupational Hazards** | | | | | | | | |
| Knowledge and Utilization of Sex-Specific Lead Aprons Among Pediatric Orthopaedic Surgeons | Journal of Pediatric Orthopedics | 2024 | Mengers et al.^153^ | F | F | USA | Survey study | Knowledge of female-specific lead is low with a lack of availability as a common barrier to utilization. |

**Table S8. Summary of Characteristics of Included Articles in the Monetary Aspects Theme**

| **Title** | **Journal** | **Year** | **Author** | **First Author Gender** | **Corresponding**  **Author Gender** | **Country** | **Study type** | **Main Study Finding** |
| --- | --- | --- | --- | --- | --- | --- | --- | --- |
| **Compensation** | | | | | | | | |
| Disparities Among Industry's Highly Compensated Orthopaedic Surgeons | JB & JS Open Access | 2021 | Robin et al.^154^ | M | F | USA | Retrospective review | Of the 347 highest-compensated orthopaedic surgeons, only 1 is a woman. |
| Orthopaedics and the gender pay gap: A systematic review | The Surgeon: Journal of the Royal Colleges of Surgeons of Edinburgh and Ireland | 2023 | Halim et al.^155^ | M | M | Great Britain | Systematic review | A gender gap exists in orthopaedics and women earn significantly less than their male colleagues for unclear reasons, even after accounting for confounding variables. |
| The Effect of Sex on Orthopaedic Surgeon Income | The Journal of Bone and Joint Surgery. American volume. | 2019 | Beebe et al.^156^ | F | M | USA | Retrospective review | Male surgeons receive higher incomes than women ($802,474 vs. $560,618) despite working equivalent hours. |
| Total knee arthroplasty reimbursement is declining overall and at a marginally faster rate amongst female orthopaedic surgeons: A Medicare analysis | Journal of Orthopaedics | 2025 | Sullivan et al.^157^ | F | F | USA | Retrospective review | Female representation among surgeons who perform TKAs is increasing; however, males treat more patients and perform more billable services than female surgeons and reimbursement disparities exist between men and women. |
| Does Merit-based Incentive Payment System Performance Differ Based on Orthopaedic Surgeon Gender? | Clinical Orthopaedics and Related Research | 2024 | Gill et al.^158^ | M | M | USA | Retrospective review | Women orthopaedic surgeons scored slightly higher on the MIPS in 2021, after controlling for surgeon and patient variables, despite providing care for a higher percentage of dual Medicare-Medicaid eligible patients and more medically complex patients. |
| **Industry Payments** | | | | | | | | |
| Men Receive Three Times More Industry Payments than Women Academic Orthopaedic Surgeons, Even After Controlling for Confounding Variables | Clinical Orthopaedics and Related Research | 2020 | Forrester et al.^159^ | F | F | USA | Retrospective review | Median payment for men surgeons was greater than women, with women academic orthopaedic surgeons receiving only 29% of the industry payments received by men. |
| Gender Disparities in Financial Relationships Between Industry and Orthopaedic Surgeons | The Journal of Bone and Joint Surgery. American volume. | 2020 | Ray et al.^160^ | F | F | USA | Retrospective review | The majority (99.6%) of royalties and consulting fees were paid to men, while only 0.4% went to women, and male gender was a predictor of total number of payments. |
| Social Media Influence and Gender Are Correlated with Industry Payments to Orthopaedic Sports Surgeons | The Journal of Knee Surgery | 2024 | Leong et al.^161^ | F | F | USA | Retrospective review | Female sports surgeons received significantly less industry non-research funding compared with their male colleagues. |
| **Grant Funding** | | | | | | | | |
| Using a Modern Linked Research Database to Examine Gender Disparities in Orthopaedic Grant Funding from 2010 to 2022 | The Journal of Bone and Joint Surgery. American volume. | 2024 | Harris et al.^162^ | M | F | USA | Retrospective review | Significantly greater grant funding was awarded to men than women, and men PIs accounted for the majority of grants received. |
| Academic career outcomes of Orthopedic Research and Education Foundation resident grant recipients | Journal of Orthopaedic Research: official publication of Orthopaedic Research Society | 2023 | Silvestre et al.^163^ | M | M | USA | Retrospective review | OREF resident grant recipients had greater women representation than the national cohort of orthopedic surgery residents. |

**Table S9. Summary of Characteristics of Included Articles in the Geographic Representation Theme**

| **Title** | **Journal** | **Year** | **Author** | **First Author Gender** | **Corresponding**  **Author Gender** | **Country** | **Study type** | **Main Study Finding** |
| --- | --- | --- | --- | --- | --- | --- | --- | --- |
| Geographic trends in the orthopedic surgery residency match | Journal of Graduate Medical Education | 2018 | Cox et al.^164^ | M | M | USA | Retrospective review | There is an association among hometown, undergraduate institution, and medical school for the training program location in which orthopedic surgery residents match, with variability in locations matched at state and census division levels. |
| Current Gender Diversity and Geographic Trends Among Orthopaedic Sports Medicine Surgeons in the United States | Orthopaedic Journal of Sports Medicine | 2022 | Kocjan et al.^165^ | F | F | USA | Cross-sectional | A greater percentage of female orthopaedic sports medicine fellows practice in the Northeastern and Midwestern regions than in the Southern and Western regions. |
| Gender Representation in Orthopaedic Surgery: A Geospatial Analysis From 2015 to 2022 | Cureus | 2022 | Peterman et al.^166^ | M | M | USA | Retrospective review | There are relative hotspots of gender diversity in the Northwest, Northeast, and Southwest, and relative cold spots of gender diversity in the Midwest and Southern U.S. |
| Geographic Differences in Sex and Racial Distributions Among Orthopaedic Surgery Residencies: Programs in the South Less Likely to Train Women and Minorities | Journal of the American Academy of Orthopaedic Surgeons. Global Research & Reviews | 2019 | Rajani et al.^167^ | M | M | USA | Retrospective review | Orthopaedic residency programs in the South had the lowest female representation, whilst programs in the West and Northeast had the highest female representation (P = 0.034). |
| Limb Lengthening and Reconstruction Society orthopedic surgeons in the United States: An analysis of geographical distribution, academic, leadership, and demographic characteristics | World Journal of Orthopedics | 2024 | Hoveidaei et al.^168^ | M | M | USA | Retrospective review | Gender disparities exist in limb lengthening and reconstruction leadership. |

**Table S10. Summary of Characteristics of Included Miscellaneous Articles**

| **Title** | **Journal** | **Year** | **Author** | **First Author Gender** | **Corresponding**  **Author Gender** | **Country** | **Study type** | **Main Study Finding** |
| --- | --- | --- | --- | --- | --- | --- | --- | --- |
| **Competency** | | | | | | | | |
| Personal Characteristics Associated with Progression in Trauma and Orthopaedic Specialty Training: A Longitudinal Cohort Study | Journal of Surgical Education | 2022 | Hope et al.^169^ | F | M | GBR | Retrospective review | Women orthopaedic trainees had a 26% increased risk of a nonstandard outcome on the Annual Review of Competency Progression (ARCP). |
| Academic Metrics Do Not Explain the Underrepresentation of Women in Orthopaedic Training Programs | Journal of Bone and Joint Surgery | 2019 | Poon et al.^170^ | F | F | USA | Retrospective review | The growth rate of the proportion of women in orthopaedic residencies lags other surgical subspecialties but appears to be independent of academic metrics. |
| Initial review of Electronic Residency Application Service charts by orthopaedic residency faculty members. Does applicant gender matter? | Journal of Bone and Joint Surgery | 2001 | Scherl et al.^171^ | F | F | USA | Experimental | The low percentage of female residents is not due to bias against female applicants in the initial chart-review phase of the orthopaedic residency selection process. |
| **Operative Autonomy** | | | | | | | | |
| A comparison of operative autonomy between men and women in orthopaedic surgical training in Aotearoa New Zealand | The New Zealand medical journal | 2023 | Bond et al.^172^ | F | F | New Zealand | Retrospective Review | While women trainees performed slightly more cases per year than men, men performed significantly more procedures unsupervised than women (45% vs. 39%). |
| The role of Gender in Operative Autonomy in orthopaedic Surgical Trainees (GOAST) | Bone and Joint Journal | 2023 | Downie et al.^173^ | F | F | Great Britain | Retrospective Review | This study showed that males perform 3% more cases as the lead surgeon than females during UK orthopaedic training (p < 0.001), and the difference was more marked during later training. |
| The correlation between trainee gender and operative autonomy during trauma and orthopaedic training in Ireland and the UK | Bone & joint open | 2025 | Mc Colgan et al.^174^ | F | M | Ireland | Retrospective Review | Men orthopaedic trainees had 145% increased odds of performing an operation with autonomy than a woman trainee. |
| **Career Plans** | | | | | | | | |
| Career plans of current orthopaedic residents with a focus on sex-based and generational differences | Journal of Bone and Joint Surgery | 2011 | Hariri et al.^175^ | F | M | USA | Survey study | More women orthopaedic residents than men planned on a subspecialty-only practice, and more planned to reduce work hours or move to part-time at some point in their careers, with implications for workforce planning. |
| Fellowship and future career plans for orthopedic trainees: gender-based differences in influencing factors | Heliyon | 2022 | Alomar^176^ | M | M | Saudi Arabia | Survey Study | Pediatric orthopedics and hand and upper extremity were top sub-specialties preferences among Saudi Arabian women trainees while arthroscopy and sports medicine, arthroplasty, and trauma were the top preferences among men. Personal interest and social and family commitments were the most influential factors for women. |
| Gender Differences in Pediatric Orthopaedics: What Are the Implications for the Future Workforce? | Clinical orthopaedics and related research | 2016 | Amoli et al.^177^ | F | M | USA | Survey Study | Among new graduates into pediatric orthopaedics, women are more likely to choose academic practice than men, providing opportunity to develop more women leaders and role models at major pediatric centres. |
|  | | | | | | | | |
| Perception of Residency Program Diversity Is Associated With Vulnerability to Race and Gender Stereotype Threat Among Minority and Female Orthopaedic Trainees | JB & JS open access | 2025 | Reid et al.^178^ | F | F | USA | Survey study | Women orthopaedic trainees had higher vulnerability to stereotype threat, especially in programs perceived as lacking racial or gender diversity. |
| Is the Distribution of Awards Gender-balanced in Orthopaedic Surgery Societies? | Clinical orthopaedics and related research | 2021 | Gerull et al.^179^ | F | F | USA | Retrospective review | In general, women received awards on par with society membership, but were more likely to receive awards in diversity or education than a leadership award. |
