# Supplementary material for: Gender Equity Issues in Orthopaedics: A Scoping Review
Source: Indian J Orthop. 2025 May 26;59(10):1609–20. doi: 10.1007/s43465-025-01415-4 (PMC12535563; doi:10.1007/s43465-025-01415-4)
Supplement: Supplementary file 4 — Supplementary file4 (DOCX 40 KB) [file 43465_2025_1415_MOESM4_ESM.docx]

**Supplemental References: Studies Included in the Systematic Review**

***Gender Diversity & Representation Theme***

1. Biermann JS. Women in orthopedic surgery residencies in the United States: Academic Medicine. 1998;73(6):708–9.
2. Van Heest AE, Fishman F, Agel J. A 5-year update on the uneven distribution of women in orthopaedic surgery residency training programs in the United States. The Journal of Bone and Joint Surgery. 2016 Aug 3;98(15):e64.
3. Poon S, Kiridly D, Mutawakkil M, Wendolowski S, Gecelter R, Kline M, et al. Current trends in sex, race, and ethnic diversity in orthopaedic surgery residency. J Am Acad Orthop Surg. 2019 Aug 15;27(16):e725–33.
4. Wang JC, Chang SW, Nwachuku I, Hill WJ, Munger AM, Suleiman LI, et al. Trends in race and sex representation among entering orthopaedic surgery residents: a continued call for active diversification efforts. J Am Acad Orthop Surg. 2023 Apr 17 ;(31(12)):e530–9.
5. Frazer A, Ndoja S, Howe HS, Singh S. Gender trends in orthopedic surgical residency programs in Canada over 20 years. Canadian Journal of Surgery. 2025 Jan 16;68(1):E41–7.
6. Haruno LS, Chen X, Metzger M, Lin CA, Little MTM, Kanim LEA, et al. Racial and sex disparities in resident attrition in orthopaedic surgery. JBJS Open Access. 2023 Apr;8(2).
7. Han Y, Fones L, Shakked R, Hammoud S, Han Y, Fones L, et al. Orthopedic surgery residency program rankings and gender diversity. Cureus. 2024 Mar 18;16(3).
8. Julian KR, Anand M, Sobel AD, Mulcahey MK, Wong SE. A 5-year update and comparison of factors related to the sex diversity of orthopaedic residency programs in the United States. JBJS Open Access. 2023 Jan;8(1).
9. Van Heest AE, Agel J, Samora JB. A 15-year report on the uneven distribution of women in orthopaedic surgery residency training programs in the United States. JBJS Open Access. 2021;6(2).
10. Van Heest AE, Agel J. The uneven distribution of women in orthopaedic surgery resident training programs in the united states: The Journal of Bone and Joint Surgery-American Volume. 2012 Jan;94(2):e9-1–8.
11. Adkins S, Hughes D, Zimmerman M, Templeton K. Correlations between department and training program online presence and women in orthopedic surgery training. Women’s Health Reports [Internet]. 2023 Mar 1;4(1):103–10.
12. Buckley JM, Dearolf LM, Wood L, Agel J, Van Heest AE, Lattanza LL. The impact of sustained outreach efforts on gender diversity in orthopaedic surgery. Journal of Bone and Joint Surgery. 2025 Jan 1;107(1):e1.
13. Cannada LK. Women in orthopaedic fellowships: what is their match rate, and what specialties do they choose? Clinical Orthopaedics & Related Research. 2016 Sep;474(9):1957–61.
14. Haddad D, Nelson D, Sherman N, Tatusko M, DeSilva G. Gender diversity in orthopaedic surgery residencies does not translate to accreditation council for graduate medical education–accredited fellowships. JBJS Open Access. 2024 Apr;9(2).
15. Poon S, Kiridly D, Brown L, Wendolowski S, Gecelter R, Vetere A, et al. Evaluation of sex, ethnic, and racial diversity across us acgme–accredited orthopedic subspecialty fellowship programs. Orthopedics. 2018 Sep;41(5):282–8.
16. Silvestre J, Kermanshahi N, Ahn J, Harris MB, Dehghan N. Factors associated with increased sex diversity in orthopaedic trauma association-accredited fellowship training. J Am Acad Orthop Surg [Internet]. 2024 Dec 10 [cited 2025 Mar 28]
17. Jabbal M, Cherry J, Eastwood D, Scott CEH, Walmsley P, Baird E. STEP 1: The Scottish Trauma & Orthopaedics Equality Project: demographics and working patterns of a national workforce. Bone & Joint Open [Internet]. 2025 Jan 21 [cited 2025 Mar 28];6(1):103–8.
18. Silvestre J, Ahn J, Dehghan N, Gitajn IL, Slobogean GP, Harris MB. Analysis of the diversity pipeline for the orthopedic trauma surgeon workforce in the United States. Injury [Internet]. 2024 Aug;55(8):111695.
19. Cho E, Bialek SE, Levack AE. Analysis of sex diversity within orthopedic trauma surgery fellowship programs. HSS Journal®: The Musculoskeletal Journal of Hospital for Special Surgery. 2024 Aug 27;15563316241272424.
20. Silvestre J, Neal T, Nelson CL, Lieberman JR, Peters CL, Chen AF. Sex diversity in the emerging united states arthroplasty workforce is limited. The Journal of Arthroplasty [Internet]. 2024 Nov;S0883540324011732.
21. Xu R, Ditelberg S, Mont MA, Olsen AS, Iorio R, Chen AF. Trends in women adult reconstruction surgeons: fellowship applicants and American association of hip and knee surgeons members. The Journal of Arthroplasty. 2025 Feb;40(2):511-515.e1.
22. Ajayi PT, Murdock CJ, Destine H, Trenchfield D, Aiyer A, Oni J. Trends in racial, ethnic, and gender diversity in orthopaedic surgery adult reconstruction fellowships from 2007 to 2021. The Journal of Arthroplasty. 2023 Nov;38(11):2232–6.
23. Cho E, Gianakos AL, Schiff AP. Analysis of gender diversity within foot and ankle surgery fellowship programs. Foot Ankle Int. 2024 Mar;45(3):272–8.
24. Joshi A, Kim A, Hsu N, Aiyer A, Thompson JM. A comparison of demographic diversity between orthopaedic surgery residents and acgme foot and ankle fellows from 2007 to 2022. Foot & Ankle Orthopaedics. 2024 Jul;9(3):24730114241263056.
25. Lavorgna TR, Gupta S, Maginnis C, Saraf SM, Stamm MA, Wong SE, et al. Persistent lack of female orthopaedic sports medicine fellows. Arthroscopy, Sports Medicine, and Rehabilitation [Internet]. 2023 Aug [cited 2025 Mar 28];5(4):100725.
26. Silvestre J, Slone HS, Newton WN, Kolade OO, Kelly JD. Sex, race, and ethnic diversity of the emerging u. S. Orthopaedic sports medicine workforce is limited. Arthroscopy: The Journal of Arthroscopic & Related Surgery. 2024 Nov;S0749806324008843.
27. Trenchfield D, Murdock CJ, Destine H, Jain A, Lord E, Aiyer A. Trends in racial, ethnic, and gender diversity in orthopedic surgery spine fellowships from 2007 to 2021. Spine. 2023 Oct 15;48(20):E349–54.
28. Meert C, Manon J, Cornu O. Female representation in orthopedic surgery: where do we stand in Belgium ? Acta Orthop Belg [Internet]. 2023 Dec [cited 2025 Mar 28];89(4):671–7.
29. Rodgers BM, Moore ML, Mead-Harvey C, Pollock JR, Thomas OJ, Beauchamp CP, et al. How does orthopaedic surgeon gender representation vary by career stage, regional distribution, and practice size? A large-database medicare study. Clin Orthop Relat Res [Internet]. 2023 Feb [cited 2025 Mar 28];481(2):359–66.
30. Shah KN, Ruddell JH, Scott B, Reid DBC, Sobel AD, Katarincic JA, et al. Orthopaedic surgery faculty: an evaluation of gender and racial diversity compared with other specialties. JBJS Open Access [Internet]. 2020 [cited 2025 Mar 28];5(3):e20.00009-e20.00009.
31. Yue T, Khosa F, Yue T, Khosa F. Academic gender disparity in orthopedic surgery in canadian universities. Cureus [Internet]. 2020 Mar 8 [cited 2025 Mar 28];12(3).
32. Kuhns B, Haws BE, Kaupp S, Maloney MD, Carmody EE, Mannava S. Academic orthopaedics as a driver of gender diversity in the orthopaedic workforce: a review of 4,519 orthopaedic faculty members. JAAOS Glob Res Rev [Internet]. 2022 Feb [cited 2025 Mar 28];6(2).
33. Acuña AJ, Sato EH, Jella TK, Samuel LT, Jeong SH, Chen AF, et al. How long will it take to reach gender parity in orthopaedic surgery in the united states? An analysis of the national provider identifier registry. Clin Orthop Relat Res [Internet]. 2021 Jun [cited 2025 Mar 28];479(6):1179–89.
34. Silvestre J, Moore M, LaPorte DM, Sabesan VJ, Van Heest A. Sex diversity and equity among fellows of the american orthopaedic association. Journal of Bone and Joint Surgery. 2025 Jan 15;107(2):222–7.
35. Oyem PC, Runsewe OI, Huffman N, Pasqualini I, Rullán PJ, Klika AK, et al. Trends in gender diversity among total hip arthroplasty surgeons. J Am Acad Orthop Surg. 2024 Dec 15;32(24):1130–7.
36. Wright MA, Aleem A, Murthi AM, Zmistowski B. Gender differences among shoulder arthroplasty surgeons: past, present, and future. Journal of Shoulder and Elbow Surgery. 2024 Aug;33(8):1799–804.
37. Gill VS, Lin E, Payne CS, Cancio-Bello A, Haglin JM, Tokish JM. Differences in primary total shoulder arthroplasty volume, reimbursement, practice styles, and patient populations based on surgeon gender: a temporal analysis. Journal of Shoulder and Elbow Surgery. 2024 Oct;S1058274624007080.
38. Lastoria DAA, Casey L, Beni R, Papanastasiou AV, Kamyab AA, Devetzis K, et al. Gender diversity in the national joint registry. Bone & Joint Open. 2024 Aug 6;5(8):637–43.
39. Schick S, Chandler K, Kasprow S, Sisk M, Elphingstone J, Wing J, et al. Gender disparities among professional team sports medicine physicians. Clinical Journal of Sport Medicine. 2023 Nov;33(6):648–51.
40. Leal J, Clifford AL, Anastasio AT, Dymtruk M, Roach RP. Diversity within the field of orthopedic sports medicine: a systematic review. JBJS Reviews. 2023 Oct;11(10).
41. Opara OA, Narayanan R, Tarawneh OH, Lee Y, Tomlak A, Zavitsanos A, et al. Race, ethnicity, and gender representation among us academic spine surgeons. J Am Acad Orthop Surg. 2025 Feb 1;33(3):e151–60.
42. Shazadeh Safavi K, Okereke R, Rezvani A, Kocjan K, Jupiter DC, Janney CF. Gender and geographic trends among foot and ankle surgeons: where are we and where do we need to improve? Foot & Ankle Specialist. 2024 Oct;17(5):431–41.
43. Daniels CM, Dworak TC, Anderson AB, Brelin AM, Nesti LJ, McKay PL, et al. Gender disparities within us army orthopedic surgery: a preliminary report. Military Medicine. 2018 Jan 1;183(1–2):e162–6.
44. Day CS, Lage DE, Ahn CS. Diversity based on race, ethnicity, and sex between academic orthopaedic surgery and other specialties: a comparative study. Journal of Bone and Joint Surgery. 2010 Oct 6;92(13):2328–35.
45. Daniels EW, French K, Murphy LA, Grant RE. Has diversity increased in orthopaedic residency programs since 1995? Clinical Orthopaedics & Related Research. 2012 Aug;470(8):2319–24.

***Research and Authorship Theme***

1. Nwosu C, Wittstein JR, Erickson MM, Schroeder N, Santiesteban L, Klifto C, et al. Representation of Female Speakers at the American Academy of Orthopaedic Surgeons Annual Meetings Over Time. JAAOS - Journal of the American Academy of Orthopaedic Surgeons. 2023 Mar 15;31(6):283.
2. Lee Y, Issa TZ, Lambrechts MJ, Carey P, Becsey A, Qadiri QS, et al. Gender disparities among speakers at major spine conferences. Spine J. 2023 Sep 9;S1529-9430(23)03372-7.
3. Mencia MM, Bidaisee S, Quan Soon C, Cawich SO. Greater Gender Diversity Observed at Orthopaedic Conferences in the Caribbean Than in the United States or England. Cureus. 2022 Aug;14(8):e28224.
4. Cohen-Rosenblum AR, Bernstein JA, Cipriano CA. Gender Representation in Speaking Roles at the American Association of Hip and Knee Surgeons Annual Meeting: 2012-2019. The Journal of Arthroplasty. 2021 Jul 1;36(7):S400–3.
5. Klein C, Pannier S, Badina A, Plancq MC, Gaumé M. Abstracts accepted for the 2021–2023 French Orthopaedic and Traumatology Society meetings: Proportion of women submitters. Orthopaedics & Traumatology: Surgery & Research. 2024 Sep 26;104007.
6. Vivekanantha P, Dao A, Hiemstra L, Shields M, Chan A, Wadey V, et al. Gender Representation in Major Orthopaedic Surgery Meetings: A Quantitative Analysis. JBJS Open Access. 2023 Dec;8(4):e23.00067.
7. Tougas C, Valtanen R, Bajwa A, Beck JJ. Gender of presenters at orthopaedic meetings reflects gender diversity of society membership. Journal of Orthopaedics. 2020 May 1;19:212–7.
8. Potter JS, Ranpura A, Rynecki ND, Beebe KS, Galdi B. Gender Parity in Academic Leadership Roles at AOSSM Annual Meetings. Orthop J Sports Med. 2021 Jan;9(1):2325967120979995.
9. Nagamine S, Morimoto T, Niizeki Y, Yamauchi K, Oizumi N, Hirata H, et al. Academic Engagement of Women as Orthopaedic Surgeons at the Annual Meetings of the Japanese Orthopaedic Association From 2012 to 2022. Cureus. 2024 Apr;16(4):e57474.
10. Gerull KM, Kim DJ, Cogsil T, Rhea L, Cipriano C. Are Women Proportionately Represented as Speakers at Orthopaedic Surgery Annual Meetings? A Cross-Sectional Analysis. Clinical Orthopaedics and Related Research®. 2020 Dec;478(12):2729.
11. Samineni A, Tornetta PI. Diversity on the American Academy of Orthopaedic Surgeons National Meeting Podium: Changes Over Two Decades. JAAOS - Journal of the American Academy of Orthopaedic Surgeons. 2024 Sep 1;32(17):793.
12. Snow M, McDaniel C, Bronheim RS, LaPorte D. Representation of women and underrepresented minorities among grand rounds speakers in orthopaedic surgery. Surgery in Practice and Science. 2022 Dec 1;11:100148.
13. Brown MA, Erdman MK, Munger AM, Miller AN. Despite Growing Number of Women Surgeons, Authorship Gender Disparity in Orthopaedic Literature Persists Over 30 Years. Clinical Orthopaedics and Related Research®. 2020 Jul;478(7):1542.
14. Rynecki ND, Krell ES, Potter JS, Ranpura A, Beebe KS. How Well Represented Are Women Orthopaedic Surgeons and Residents on Major Orthopaedic Editorial Boards and Publications? Clinical Orthopaedics and Related Research®. 2020 Jul;478(7):1563.
15. Hiller KP, Boulos A, Tran MM, Cruz AIJ. What Are the Rates and Trends of Women Authors in Three High-impact Orthopaedic Journals from 2006-2017? Clinical Orthopaedics and Related Research®. 2020 Jul;478(7):1553.
16. Seetharam A, Ali MT, Wang CY, Schultz KE, Fischer JP, Lunsford S, et al. Authorship trends in the Journal of Orthopaedic Research: A bibliometric analysis. J Orthop Res. 2018 Nov;36(11):3071–80.
17. Okike K, Liu B, Lin YB, Torpey JL, Kocher MS, Mehlman CT, et al. The orthopedic gender gap: trends in authorship and editorial board representation over the past 4 decades. Am J Orthop (Belle Mead NJ). 2012 Jul;41(7):304–10.
18. Vitale E, Bizzoca D, Di Dio F, Moretti A, Moretti B. The gender role in the publishing of Authorships in high-impact orthopedic journals. Musculoskelet Surg. 2024 Sep 1;108(3):289–95.
19. Saka N, Chiang CM, Ogawa T, Pendleton AA, Tsuihiji K, Nomura K, et al. Trend of female first authorship in Journal of Orthopaedic Science, the official journal of the Japanese orthopaedic association from 2001 to 2021: An observational study. J Orthop Sci. 2024 May;29(3):914–20.
20. Grant C, Stauffer TP, Seyler TM, Wu CJ, Hinton ZW. Gender Trends in Authorship in 6 Major Orthopaedic Journals. JBJS. 2024 Apr 3;106(7):625.
21. Khalifa AA, El-Hawary AS, Sadek AE, Ahmed EM, Ahmed AM, Haridy MA. Comparing the gender diversity and affiliation trends of the authors for two orthopaedics journals from the Arab world. J Taibah Univ Med Sci. 2021 Feb;16(1):1–8.
22. Ghattas YS, Kyin C, Grise A, Glasser J, Johnson T, Druskovich K, et al. Trends in Female Authorship in Orthopaedic Literature from 2002 to 2021: An Analysis of 168,451 Authors. JBJS. 2023 Aug 16;105(16):1285.
23. Okewunmi J, Kiani SN, Poeran J, Galatz LM. Female Authorship in the US Orthopaedics Literature: A Bibliometric Analysis of Trends. JAAOS - Journal of the American Academy of Orthopaedic Surgeons. 2023 Jun 15;31(12):627.
24. Preut J, Frosch KH, Debus ES, Grundmann RT. Bibliometric Analysis of Research Areas, Publication Hierarchy and Gender Authorship in German University Orthopaedic Surgery. Z Orthop Unfall. 2023 Oct;161(5):516–25.
25. Powell SN, Hunting JC, Frazier LP, Keeling LE, Janowski J. Evolution and Trends in Male Versus Female Authorship of Articles in Flagship Orthopaedic Journals From 1995 to 2020. JAAOS - Journal of the American Academy of Orthopaedic Surgeons. 2022 Jun 15;30(12):e878.
26. Prior A, Ogburu-Ogbonnaya N, Barfield WR, Mooney JFI, Van Nortwick S, Murphy RF. Analysis of Author Gender in the Pediatric Orthopaedic Literature from 2011 to 2020. Journal of Pediatric Orthopaedics. 2021 Aug;41(7):e481.
27. Johnson MA, Mulvey H, Parambath A, Anari JB, Wall LB, Shah AS. A Gender Gap in Publishing? Understanding the Glass Ceiling in Pediatric Orthopaedic Surgery. Journal of Pediatric Orthopaedics. 2021 Aug;41(7):e484.
28. Videckis AJ, Malyavko A, Kraft DB, Tabaie SA. Male Versus Female Authorship in Flagship Pediatric Orthopaedic Journals From 2002 to 2021. Journal of Pediatric Orthopaedics. 2023 Jul;43(6):e493.
29. Xu RF, Varady NH, Chen AF. Disparities Among Leading Publishers of Arthroplasty Research. The Journal of Arthroplasty. 2021 May 1;36(5):1804–9.
30. Xu RF, Varady NH, Chen AF. Trends in Gender Disparities in Authorship of Arthroplasty Research. JBJS. 2020 Dec 2;102(23):e131.
31. Watters AB, Blitz J, Mortell T, Ierulli VK, Lefante J, Mulcahey MK. A 15-Year Bibliometric Analysis of Sports Medicine Studies in The Journal of Bone and Joint Surgery: A Systematic Review. JBJS Open Access. 2024 Dec;9(4):e24.00045.
32. Kim CY, Sivasundaram L, Trivedi NN, Gilmore A, Gillespie RJ, Salata MJ, et al. A 46-year Analysis of Gender Trends in Academic Authorship in Orthopaedic Sports Medicine. JAAOS - Journal of the American Academy of Orthopaedic Surgeons. 2019 Jul 1;27(13):493.
33. Russell AF, Nguyen M, Bhuiya M, Likine EF, Fischer JP, Grassel K, et al. Comparative Analysis of Bibliometric, Authorship, and Collaboration Trends Over the Past 30-Year Publication History of the Journal of Orthopaedic Trauma and Injury. Journal of Orthopaedic Trauma. 2018 Aug;32(8):e327.
34. Benes G, ElNemer W, Avendano J, Hsu N, Aiyer A. Research Productivity and Impact in Foot and Ankle Surgery: Insights From Relative Citation Ratio Analysis of Recent Fellowship Graduates. J Am Acad Orthop Surg Glob Res Rev. 2024 Feb 1;8(2):e23.00280.
35. Fanfan D, Larios F, Gonzalez MR, Rodriguez A, Nichols D, Alvarez JC, et al. A Bibliometric Analysis of the 500 Most Cited Papers in Orthopaedic Oncology. J Am Acad Orthop Surg Glob Res Rev. 2024 Jan 1;8(1):e23.00223.
36. Sequeira SB, Wright MA, Murthi AM. Gender disparities in shoulder and elbow publications. Journal of Shoulder and Elbow Surgery. 2022 Dec 1;31(12):e613–9.
37. Ellsworth BK, Pascual-Leone N, Gross PW, Barth KA, Doyle SM. Is There Gender Disparity in Orthopedic Surgery Resident Research Productivity? HSS Journal®. 2024 May 1;20(2):274–81.
38. Cho E, McCarthy MV, Hodkiewicz V, Rumps MV, Mulcahey MK. Gender Disparity in Authorship Among Orthopaedic Surgery Residents. JBJS Open Access. 2024 Sep;9(3):e24.00061.
39. Silvestre J, Walker JJ, LaPorte DM, Nelson CL. Women Are Underrepresented Among Principal Investigators of Hip and Knee Arthroplasty Clinical Trials in the United States. JBJS. 2023 Nov 1;105(21):1734.
40. Burkhart RJ, Karimi AH, Hecht CJI, Avila A, Acuña AJ, Kamath AF. What Are the Trends in Women’s Representation Among Lead Investigators of Orthopaedic Clinical Trials? Clinical Orthopaedics and Related Research®. 2024 Jan;482(1):35.
41. Ozdag Y, Luciani AM, Foster BK, Baylor JL, Hayes DS, Gabelus S, et al. Orthopaedic Research Consortiums: A Review of Scope, Sex and Racial Representation. Cureus. 2024 Mar;16(3):e55859.

***Leadership & Mentorship Theme***

1. Ranson R, Webber K, Saker C, Cashin I, Bunstine JL, Patel AP, et al. Representation matters: a higher percentage of women orthopaedic surgery faculty is associated with an increased number of women residents. J Am Acad Orthop Surg. 2025 Mar 1;33(5):253–60.
2. Sobel AD, Cox RM, Ashinsky B, Eberson CP, Mulcahey MK. Analysis of factors related to the sex diversity of orthopaedic residency programs in the united states. The Journal of Bone and Joint Surgery. 2018 Jun 6;100(11):e79.
3. Hill JF, Yule A, Zurakowski D, Day CS. Residents’ perceptions of sex diversity in orthopaedic surgery. Journal of Bone and Joint Surgery. 2013 Oct 2;95(19):e144.
4. Winfrey SR, Parameswaran P, Gaerull KM, LaPorte D, Cipriano CA. Effective mentorship of women and underrepresented minorities in orthopaedic surgery: a mixed-methods investigation. JBJS Open Access. 2022 Oct;7(4).
5. Ramírez C, Iñiguez M, Reginato R, Ibáñez A, Ahumada X. Satisfacción personal y percepción de éxito en mujeres en Traumatología: Factores determinantes en la elección de la especialidad y desarrollo profesional. Revista Chilena de Ortopedia y Traumatología. 2022 Dec [cited 2025 Mar 28];63(03):e145–9.
6. Dias R, Herzog I, Alomary S, Beebe KS. Is program director gender associated with gender diversity among orthopaedic surgery residency programs? Clin Orthop Relat Res. 2024 Aug;482(8):1351–7.
7. Hill JF, Johnson AH, Cannada L. A profile of female academic orthopaedic surgeons. Current Orthopaedic Practice. 2013 Nov;24(6):636–40.
8. Budin JS, Rumps MV, Mulcahey MK. Sex, race, and ethnicity of faculty and department chairs in orthopaedic surgery and comparable fields: 2015 to 2022. J Am Acad Orthop Surg. 2024 Dec 1;32(23):1108–14.
9. Chen RE, Kuhns BD, Kaupp S, Voloshin I, Mannava S. Diversity among academic orthopedic shoulder and elbow surgery faculty in the United States. Journal of Shoulder and Elbow Surgery. 2020 Apr;29(4):655–9.
10. Asturias AM, Wague A, Feeley LA, Senter C, Pandya N, Feeley BT, et al. Gender disparities in endowed professorships within orthopaedic surgery. Cureus. 2024 Feb 28;16(2).
11. Moore ML, Elahi MA, Doan MK, Pollock JR, Makovicka JL, Hassebrock JD, et al. Orthopaedic sports medicine fellowship directors are predominantly white men with a high degree of research productivity. Arthroscopy, Sports Medicine, and Rehabilitation. 2021 Oct;3(5):e1449–55.
12. Kamalapathy PN, Raso J, Rahman R, Harihar S, Lozano-Calderon S, Hassanzadeh H. Orthopedic surgery fellowship directors: trends in demographics, education, employment, and institutional familiarity. HSS Journal®: The Musculoskeletal Journal of Hospital for Special Surgery. 2023 Feb;19(1):113–9.
13. Cummings PE, Alder KD, Marigi EM, Hidden KA, Kakar S, Barlow JD. Demographics and characteristics of orthopaedic surgery residency program directors: a cross-sectional review. JBJS Open Access. 2023 Jan;8(1).
14. Meadows AM, Skinner MM, Faraj MT, Hazime AA, Day RG, Fore JA, et al. Racial, ethnic, and gender diversity in academic orthopaedic surgery leadership. Journal of Bone and Joint Surgery. 2022 Jul 6;104(13):1157–65.
15. Hunter J, Grewal R, Nam D, Lefaivre KA. Gender disparity in academic orthopedic programs in Canada: a cross-sectional study. Canadian Journal of Surgery. 2022 Mar 9;65(2):E159–69.
16. Bi AS, Fisher ND, Bletnitsky N, Rao N, Egol KA, Karamitopoulos M. Representation of women in academic orthopaedic leadership: where are we now? Clin Orthop Relat Res. 2022 Jan;480(1):45–56.
17. Proal JD, DiStefano D, Park A, Ikpeze T, Li X, Mesfin A. Demographic and academic characteristics of orthopaedic shoulder and elbow division chiefs in the United States. Journal of Shoulder and Elbow Surgery . 2025 Jan;34(1):136–40.
18. Silvestre J, Tippabhatla A, Chopra A, Nelson CL, LaPorte DM. Sex disparities among fellowship program directors in orthopaedic surgery. Journal of Bone and Joint Surgery. 2024 Feb 7;106(3):251–7.
19. Poon S, Abzug J, Caird M, Cho RH, Luong M, Weiss JM. A five-year review of the designated leadership positions of pediatric orthopaedic society of north america. Orthopedic Clinics of North America. 2019 Jul;50(3):331–5.
20. Hiemstra LA, Wittman T, Mulpuri K, Vezina C, Kerslake S. Dissecting disparity: improvements towards gender parity in leadership and on the podium within the Canadian Orthopaedic Association. Journal of ISAKOS . 2019 Sep;4(5):227–32.
21. Chrea B, Johnson H, Baumhauer J, Holleran A, Atwater LC, Poon S. A 10-year review of designated leadership positions of the american orthopaedic foot & ankle society(Aofas). Foot & Ankle Orthopaedics. 2022 Oct;7(4):24730114221133392.
22. Murphy L, Miller AN, Vallier HA, Roffey DM, Lefaivre KA. Gender diversity, leadership, promotion, and opportunity among the members of the orthopaedic trauma association(Ota). Journal of Orthopaedic Trauma. 2023 Jun;37(6):e240–6.
23. Albright P, Banks E, Wood L, Chambers C, Van Heest A. Orthopaedic society leadership diversity and academic participation: where do we stand now? Journal of Bone and Joint Surgery. 2022 Dec 21;104(24):e103.
24. Ramos T, Daban R, Kale N, Brown S, Miskimin C, Cannada LK, et al. Women in leadership in state and regional orthopaedic societies. JAAOS Glob Res Rev. 2022 Apr;6(4).
25. Saxena S, Cannada LK, Weiss JM. Does the proportion of women in orthopaedic leadership roles reflect the gender composition of specialty societies? Clin Orthop Relat Res. 2020 Jul;478(7):1572–9.
26. Tanguilig G, Meyers J, Ierulli VK, Hiemstra L, Mulcahey MK. Women in leadership in orthopaedic sports medicine societies throughout the world. Journal of ISAKOS. 2024 Jun;9(3):438–43.
27. Attia AC, Brown SM, Ladd AL, Mulcahey MK. Representation of male and female orthopedic surgeons in specialty societies. Orthopedics. 2021 Sep;44(5):289–92.
28. Steele M, Gianakos AL, Stamm MA, Mulcahey MK. Diversity in orthopaedic sports medicine societies. Arthroscopy, Sports Medicine, and Rehabilitation. 2023 Aug;5(4):100752.
29. Pujari A, Johnson F, Little MT, Forsh DA, Okike K. Racial/ethnic and gender diversity of orthopaedic journal editorial boards. Journal of Bone and Joint Surgery. 2024 Mar 6;106(5):460–5.
30. Wood R, Perera J, Krumrey J, McCrum C, Wood R, Perera J, et al. Orthopedic team surgeons in major professional sports: an analysis of affiliation with the top 10 sports medicine fellowship programs and implications for leadership and diversity. Cureus. 2024 Feb 15;16(2).

***Microaggressions & Lived Experiences Theme***

1. Halim UA, Elbayouk A, Ali AM, Cullen CM, Javed S. The prevalence and impact of gender bias and sexual discrimination in orthopaedics, and mitigating strategies: a systematic review. The Bone & Joint Journal. 2020 Nov 1;102-B(11):1446–56.
2. Alhammadi NA, Jabbar IA, Alahmari SA, Alqahtani RM, Alhadi WA, Alnujaymi BM, et al. Gender-Related Microaggressions in Orthopedic Surgery: A Comprehensive Survey of Women Orthopedists and Implications for Progress, Saudi Arabia. JHL. 2024 Jan 8;16:29–37.
3. Carino Mason MR, Pandya S, Joshi P, Cai N, Murdock CJ, Hui-Chou HG. Perceptions of Racial and Gender Microaggressions in an Academic Orthopaedic Department. JBJS Open Access. 2023 Sep;8(3):e22.00150.
4. Sobel AD, Lavorgna TR, Ames SE, Templeton KJ, Mulcahey MK, Group and the COER. Interpersonal Interactions and Biases in Orthopaedic Surgery Residency: Do Experiences Differ Based on Gender? Clinical Orthopaedics and Related Research®. 2023 Feb;481(2):369.
5. Whicker E, Williams C, Kirchner G, Khalsa A, Mulcahey MK. What Proportion of Women Orthopaedic Surgeons Report Having Been Sexually Harassed During Residency Training? A Survey Study. Clinical Orthopaedics and Related Research®. 2020 Nov;478(11):2598.
6. Giglio V, Schneider P, Bond Z, Madden K, McKay P, Bozzo A, et al. Prevalence of gender-based and sexual harassment within orthopedic surgery in Canada. Canadian Journal of Surgery. 2022 Jan 27;65(1):E45–51.
7. Samora JB, Denning J, Haralabatos S, Luong M, Poon S. Do women experience microaggressions in orthopaedic surgery? Current state and future directions from a survey of women orthopaedists. Current Orthopaedic Practice. 2020 Oct;31(5):503.
8. Jurenovich KM, Cannada LK. Women in Orthopedics and their Fellowship Choice: What Influenced their Specialty Choice? Iowa Orthop J. 2020;40(1):13–7.
9. Koschmeder KT, Hurley-Novatny AC, Marti AA, Sharp KM, Linderman SE, Coffman AR, et al. A Cross-Sectional Study of Gender-Specific Influences of Orthopedic Subspecialty Selection. Iowa Orthop J. 2024;44(1):1–10.
10. Leape CP, Hawken JB, Geng X, Wright MA, Murthi AM. An investigation into gender bias in the evaluation of orthopedic trainee arthroscopic skills. Journal of Shoulder and Elbow Surgery. 2022 Nov 1;31(11):2402–9.
11. Brady JM, Bray A, Kim P, Schneider B, Lippe J, Mercer D, et al. Female Residents Give Themselves Lower Scores Than Male Colleagues and Faculty Evaluators on ACGME Milestones. J Surg Educ. 2021;78(4):1305–11.
12. Goldstein SD, Klosterman EL, Hetzel SJ, Grogan BF, Williams KL, Guiao R, et al. The Effect of an Orthopaedic Surgeon’s Attire on Patient Perceptions of Surgeon Traits and Identity: A Cross-Sectional Survey. JAAOS Global Research & Reviews. 2020 Aug;4(8):e20.00097.
13. Peck CJ, Schmidt SJ, Latimore DA, O’Connor MI. Chair Versus Chairman: Does Orthopaedics Use the Gendered Term More Than Other Specialties? Clinical Orthopaedics and Related Research®. 2020 Jul;478(7):1583.
14. Freeman C, Evans R, Drever N, White J, Larkins S, Morrey C. Barriers and facilitators for female practitioners in orthopaedic training and practice: a scoping review. ANZ J Surg. 2025 Jan 3;
15. Lancaster AJ, Stevenson KL, Noel PH, Grothaus OF, Blackburn BE, Gililland JM. Motivations and Barriers for Women Orthopaedic Surgeons Considering Arthroplasty Fellowship. The Journal of Arthroplasty. 2024 Feb 1;39(2):527–32.
16. Xu AL, Humbyrd CJ, De Mattos CBR, LaPorte D. The Importance of Perceived Barriers to Women Entering and Advancing in Orthopaedic Surgery in the US and Beyond. World Journal of Surgery. 2023;47(12):3051–9.
17. Liew S, Lee J, Tamam F, Ismail I, Mohamed-Saaid F, Chye P. Women in Orthopaedics: A Perspective from Malaysian Female Orthopaedic Surgeons. Malays Orthop J. 2023 Mar;17(1):70–8.
18. Lieberman EG, Gerull KM, Chen AF, Bernstein JA, Cohen-Rosenblum AR, Tsao AK, et al. Factors That Influence Orthopedic Women Residents’ Selection of Adult Reconstruction. The Journal of Arthroplasty. 2023 Sep 1;38(9):1877–84.
19. Hiemstra LA, Kerslake S, Clark M, Temple-Oberle C, Boynton E. Experiences of Canadian Female Orthopaedic Surgeons in the Workplace: Defining the Barriers to Gender Equity. JBJS. 2022 Aug 17;104(16):1455.
20. Rohde RS, Wolf JM, Adams JE. Where Are the Women in Orthopaedic Surgery? Clin Orthop Relat Res. 2016 Sep;474(9):1950–6.
21. Gerull KM, Klein SE, Miller AN, Cipriano CA. Do Women and Minority Orthopaedic Residents Report Experiencing Worse Well-being and More Mistreatment Than Their Peers? Clinical Orthopaedics and Related Research®. 2024 Aug;482(8):1325.
22. Amaral A, Calcado I, Gomez A, et al. (May 29, 2024) The Perspective of Brazilian Women Orthopaedic Surgeons on Gender Discrimination: Initial Insights to Understand Gender Bias in the Brazilian Healthcare System. Cureus 16(5): e61325. doi:10.7759/cureus.61325
23. Rodarte P, Kammire MS, Israel H, Poon SC, Cannada LK. The other side of conflict: Examining the challenges of female orthopaedic surgeons in the workplace. The American Journal of Surgery. 2023 Jan 1;225(1):46–52.
24. Ponce B, Gruenberger E, McGwin G, Samora J, Patt J. Workplace Violence in Orthopaedic Surgery: A Survey of Academy of Orthopaedic Surgeons Membership. JAAOS - Journal of the American Academy of Orthopaedic Surgeons. 2024 Apr 15;32(8):e359.

***Gender-based Health Impacts Theme***

1. Kermanshahi N, Hartman H, Matzkin E, Gianakos AL. Pregnancy and infertility in orthopedics: A review of the current state. World J Surg. 2024 May;48(5):1025–36.
2. Kontoghiorghe C, Morgan C, Eastwood D, McNally S. UK pregnancy in orthopaedics (UK-POP): a cross-sectional study of UK female trauma and orthopaedic surgeons and their experiences of pregnancy. Bone Jt Open. 2023 Dec 19;4(12):970–9.
3. Morrison LJ, Abbott AG, Mack Z, Schneider P, Hiemstra LA. What Are the Challenges Related to Family Planning, Pregnancy, and Parenthood Faced by Women in Orthopaedic Surgery? A Systematic Review. Clin Orthop Relat Res. 2023 Jul 1;481(7):1307–18.
4. Wynn M, Caldwell L, Kowalski H, Lawler E. Identifying Barriers: Current Breastfeeding Policy in Orthopedic Surgery Residency. Iowa Orthop J. 2021;41(1):5–9.
5. Poon S, Luong M, Hargett D, Lorimer S, Nguyen C, Payares M, et al. Does a Career in Orthopaedic Surgery Affect a Woman’s Fertility? J Am Acad Orthop Surg. 2021 Mar 1;29(5):e243–50.
6. Mulcahey MK, Nemeth C, Trojan JD, OʼConnor MI. The Perception of Pregnancy and Parenthood Among Female Orthopaedic Surgery Residents. J Am Acad Orthop Surg. 2019 Jul 15;27(14):527–32.
7. Ruse S, Bergman R, Crawford E. Pregnancy in Orthopaedic Residents: Peripartum Barriers Identified. JB JS Open Access. 2022;7(4):e22.00098.
8. Morgan C, Li L, Kasetti PR, Varma R, Liddle AD. Pregnancy, parenthood, and fertility in the orthopaedic surgeon. Bone Joint J. 2023 Aug 1;105-B(8):857–63.
9. Frenkel Rutenberg T, Daglan E, Shadmi N, Iordache SD, Kosashvili Y, Eylon S. Fertility and pregnancy complications in female orthopaedic surgeons. Occup Med (Lond). 2024 Sep 23;74(6):403–8.
10. Hiemstra LA, Kerslake S, Fritz JA, Clark M, Temple-Oberle C, Boynton E, et al. Rates of Burnout in Female Orthopaedic Surgeons Correlate with Barriers to Gender Equity. JBJS. 2023 Jun 7;105(11):849.
11. Carter CW, Talwalkar V, Weiss JM, Schwend RM, Goldberg MJ. Pediatric Orthopaedists Are Not Immune: Characterizing Self-reported Burnout Rates Among POSNA Members. Journal of Pediatric Orthopaedics. 2020 Jul;40(6):e527.
12. Sedani AB, Yakkanti RR, Syros A, Swonger RM, LaPorte DM, Aiyer AA, et al. An overview of occupational injuries among female orthopaedic surgeons. Journal of Orthopaedics. 2024 Jan 1;47:94–9.
13. Mengers S, Emara A, Maheshwer B, Lopresti J, Mistovich RJ. Knowledge and Utilization of Sex-Specific Lead Aprons Among Pediatric Orthopaedic Surgeons. J Pediatr Orthop. 2024 Oct 1;44(9):e858–62.

***Monetary Aspects Theme***

1. Robin JX, Murali S, Paul KD, Kofskey AM, Wilson AL, Almaguer AM, et al. Disparities Among Industry’s Highly Compensated Orthopaedic Surgeons. JB JS Open Access. 2021;6(4):e21.00015.
2. Halim UA, Qureshi A, Dayaji S, Ahmad S, Qureshi MK, Hadi S, et al. Orthopaedics and the gender pay gap: A systematic review. Surgeon. 2023 Oct;21(5):301–7.
3. Beebe KS, Krell ES, Rynecki ND, Ippolito JA. The Effect of Sex on Orthopaedic Surgeon Income. J Bone Joint Surg Am. 2019 Sep 4;101(17):e87.
4. Sullivan G, Gill V, Lin EA, Cancio-Bello A, Haglin J, Bingham JS. Total knee arthroplasty reimbursement is declining overall and at a marginally faster rate amongst female orthopaedic surgeons: A Medicare analysis. J Orthop. 2025 May;63:8–15.
5. Gill VS, Lin E, Holle A, Haglin JM, Clarke HD. Does Merit-based Incentive Payment System Performance Differ Based on Orthopaedic Surgeon Gender? Clin Orthop Relat Res. 2024 Dec 20;
6. Forrester LA, Seo LJ, Gonzalez LJ, Zhao C, Friedlander S, Chu A. Men Receive Three Times More Industry Payments than Women Academic Orthopaedic Surgeons, Even After Controlling for Confounding Variables. Clin Orthop Relat Res. 2020 Jul;478(7):1593–9.
7. Ray GS, Lechtig A, Rozental TD, Bernstein DN, Merchan N, Johnson AH. Gender Disparities in Financial Relationships Between Industry and Orthopaedic Surgeons. JBJS. 2020 Feb 19;102(4):e12.
8. Leong NL, Morcos G, Jiang J, O’Hara N. Social Media Influence and Gender Are Correlated with Industry Payments to Orthopaedic Sports Surgeons. J Knee Surg. 2024 Mar;37(4):275–81.
9. Harris AB, Benes G, Ghanem D, Cartagena-Reyes M, Jain A, Laporte DM. Using a Modern Linked Research Database to Examine Gender Disparities in Orthopaedic Grant Funding from 2010 to 2022. JBJS. 2024 Jan 3;106(1):39.
10. Silvestre J, Burgess RK, Nelson CL, Thompson TL. Academic career outcomes of Orthopedic Research and Education Foundation resident grant recipients. J Orthop Res. 2023 Feb;41(2):459–65.

***Geographic Representation Theme***

1. Cox RM, Sobel AD, Biercevicz A, Eberson CP, Mulcahey MK. Geographic Trends in the Orthopedic Surgery Residency Match. J Grad Med Educ. 2018 Aug 2;10(4):423–8.
2. Kocjan K, Safavi KS, Flaherty B, Rezvani A, Jupiter DC, Janney CF, et al. Current Gender Diversity and Geographic Trends Among Orthopaedic Sports Medicine Surgeons in the United States. Orthop J Sports Med. 2022 Nov 28;10(11):23259671221134091.
3. Peterman NJ, Macinnis B, Stauffer K, Mann R, Yeo EG, Carpenter K. Gender Representation in Orthopaedic Surgery: A Geospatial Analysis From 2015 to 2022. Cureus. 14(7):e27305.
4. Rajani R, Haghshenas V, Abalihi N, Tavakoli EM, Zelle BA. Geographic Differences in Sex and Racial Distributions Among Orthopaedic Surgery Residencies: Programs in the South Less Likely to Train Women and Minorities. JAAOS Global Research & Reviews. 2019 Feb;3(2):e004.
5. Hoveidaei AH, Niakan R, Hosseini-Asl SH, Annasamudram A, Conway JD. Limb Lengthening and Reconstruction Society orthopedic surgeons in the United States: An analysis of geographical distribution, academic, leadership, and demographic characteristics. World J Orthop. 2024 Feb 18;15(2):147–55.

***Miscellaneous***

1. Hope C, Humes D, Griffiths G, Lund J. Personal characteristics associated with progression in trauma and orthopaedic specialty training: a longitudinal cohort study. Journal of Surgical Education. 2022 Jan;79(1):253–9.
2. Poon S, Nellans K, Crabb RAL, Rothman A, Wendolowski SF, Kiridly D, et al. Academic metrics do not explain the underrepresentation of women in orthopaedic training programs. The Journal of Bone and Joint Surgery. 2019 Apr 17;101(8):e32.
3. Scherl SA, Lively N, Simon MA. Initial review of electronic residency application service charts by orthopaedic residency faculty members: does applicant gender matter? The Journal of Bone and Joint Surgery-American Volume. 2001 Jan;83(1):65–70.
4. Bond EC, Whiting FH, Larsen PD, Chan G. A comparison of operative autonomy between men and women in orthopaedic surgical training in Aotearoa New Zealand. N Z Med J. 2023 Jul 21;136(1579):36-48.
5. Downie S, Cherry J, Dunn J, Harding T, Eastwood D, Gill S, et al. The role of Gender in Operative Autonomy in orthopaedic Surgical Trainees (Goast): a national collaborative project. The Bone & Joint Journal. 2023 Jul 1;105-B(7):821–32.
6. Colgan RM, Boland F, Sheridan GA, Colgan G, Bose D, Eastwood DM, et al. The correlation between trainee gender and operative autonomy during trauma and orthopaedic training in Ireland and the UK. Bone & Joint Open. 2025 Jan 11;6(1):62–73.
7. Hariri S, York SC, O’Connor MI, Parsley BS, McCarthy JC. Career plans of current orthopaedic residents with a focus on sex-based and generational differences. Journal of Bone and Joint Surgery. 2011 Mar 2;93(5):e16.
8. Alomar AZ. Fellowship and future career plans for orthopedic trainees: gender-based differences in influencing factors. Heliyon. 2022 Sep;8(9):e10597.
9. Amoli MA, Flynn JM, Edmonds EW, Glotzbecker MP, Kelly DM, Sawyer JR. Gender differences in pediatric orthopaedics: what are the implications for the future workforce? Clinical Orthopaedics & Related Research. 2016 Sep;474(9):1973–8.
10. Reid RT, Odum SM, Rosopa PJ, Brooks JT, Scannell BP, Poon S, et al. Perception of residency program diversity is associated with vulnerability to race and gender stereotype threat among minority and female orthopaedic trainees. JBJS Open Access. 2025 Jan;10(1).
11. Gerull KM, Holten A, Rhea L, Cipriano C. Is the distribution of awards gender-balanced in orthopaedic surgery societies? Clin Orthop Relat Res. 2021 Jan;479(1):33–43.
